# Chimeric Exosomes Functionalized with STING Activation for Personalized Glioblastoma Immunotherapy

**DOI:** 10.1002/advs.202306336

**Published:** 2023-12-10

**Authors:** Peng Bao, Hui‐Yun Gu, Jing‐Jie Ye, Jin‐Lian He, Zhenlin Zhong, Ai‐Xi Yu, Xian‐Zheng Zhang

**Affiliations:** ^1^ Key Laboratory of Biomedical Polymers of Ministry of Education & Department of Chemistry Wuhan University Wuhan 430072 P. R. China; ^2^ Department of Orthopedic Trauma and Microsurgery Zhongnan Hospital of Wuhan University Wuhan 430071 P. R. China

**Keywords:** chimeric exosomes, glioblastoma, personalized immunotherapy, STING activation, T cell responses

## Abstract

A critical challenge of existing cancer vaccines is to orchestrate the demands of antigen‐enriched furnishment and optimal antigen‐presentation functionality within antigen‐presenting cells (APCs). Here, a complementary immunotherapeutic strategy is developed using dendritic cell (DC)‐tumor hybrid cell‐derived chimeric exosomes loaded with stimulator of interferon genes (STING) agonists (DT‐Exo‐STING) for maximized tumor‐specific T‐cell immunity. These chimeric carriers are furnished with broad‐spectrum antigen complexes to elicit a robust T‐cell‐mediated inflammatory program through direct self‐presentation and indirect DC‐to‐T immunostimulatory pathway. This chimeric exosome‐assisted delivery strategy possesses the merits versus off‐the‐shelf cyclic dinucleotide (CDN) delivery techniques in both the brilliant tissue‐homing capacity, even across the intractable blood–brain barrier (BBB), and the desired cytosolic entry for enhanced STING‐activating signaling. The improved antigen‐presentation performance with this nanovaccine‐driven STING activation further enhances tumor‐specific T‐cell immunoresponse. Thus, DT‐Exo‐STING reverses immunosuppressive glioblastoma microenvironments to pro‐inflammatory, tumoricidal states, leading to an almost obliteration of intracranial primary lesions. Significantly, an upscaling option that harnesses autologous tumor tissues for personalized DT‐Exo‐STING vaccines increases sensitivity to immune checkpoint blockade (ICB) therapy and exerts systemic immune memory against post‐operative glioma recrudesce. These findings represent an emerging method for glioblastoma immunotherapy, warranting further exploratory development in the clinical realm.

## Graphical abstract

A complementary immunotherapeutic platform is constructed using DC‐tumor chimeric exosomes cooperated with exogenous CDN‐driven STING activation for maximized tumor‐specific T‐cell immunity. These chimeric nanovesicles provide advantages versus existing CDN delivery technologies in 1) the supply of broad‐spectrum tumor antigen complexes for dual T‐cell activation; 2) the cytosolic delivery of STING agonists for enhanced antigen presentation; and 3) the brilliant BBB‐crossing capacity.

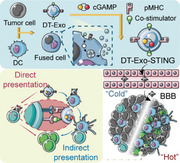

## 1 Introduction

Restoring the autologous immune system to impede treatment‐refractory malignant cancer progression, is emerging as a promising option for oncotherapy.^[^ ^1^, ^2^ ^]^ Both the potency and durability in antineoplastic immunotherapy hinge to a large extent on the activation of tumor antigen‐specific CD8^+^ T lymphocytes, which necessitates a reliance on hyper‐immunogenic antigenic stimulus.^[^ ^3^, ^4^, ^5^ ^]^ The rapid advance of the exome sequencing technique has spawned massive research of one or several designated antigen peptide‐based vaccines, widening the repertoire of tumor‐specific cytotoxicity in cancer patients.^[^ ^6^, ^7^, ^8^, ^9^ ^]^ While promising, most universal antigen‐based vaccines on trial are limited by tumor heterogeneity and thus are somewhat mediocre from the registered objective response rates.^[^ ^10^, ^11^ ^]^ To cap it all, persistent exposure to repetitive antigenic stimulation can accelerate the conversion of cytotoxic T cells to a “terminally exhausted” T‐cell phenotype, leading to immune escape and even immunologic tolerance.^[^ ^12^ ^]^ In this context, the key to developing next‐generation antigen‐targeted cancer vaccines may be inclined to construct an upscaling platform for fulfilling the demands of multi‐antigenic co‐delivery.

A paradigm that furnishes multiple antigens is to resort to tumor‐derived exosomes (T‐Exos), which harbor a broad‐spectrum antigenic formula inherited from parental cells.^[^ ^13^, ^14^, ^15^ ^]^ Whereas, they also acquire the crafty tricks of immune escape from tumor cells, and participate in enforcing a multifaceted immunosuppressive procedure.^[^ ^16^ ^]^ These fatal weaknesses of exosomes from tumor cells compel multiantigen‐oriented research bias toward seeking a successor with optimized antigen‐stimulating functionality. Given the mechanism of exosome production, modulating the characteristics of parental cells can impart their secreted exosomes with desired biological properties, which can be utilized to improve the multiantigen‐loaded nanovaccines.^[^ ^17^, ^18^, ^19^ ^]^ A similar broad‐spectrum antigen‐containing role has also been observed in a class of cellular vaccines, which generates from the fusion between antigen‐presenting cells (APCs) and tumor cells and integrates bi‐parental lineages.^[^ ^20^, ^21^, ^22^, ^23^ ^]^ Unlike the immunosuppressive molecule‐expressing T‐Exos, chimeric exosomes from these hybrid cells still carry their parental cell‐specific signatures, allowing for both peptide‐major histocompatibility complex (pMHC) derived from broad‐spectrum tumor antigens and APC‐­derived costimulatory molecules (e.g., B7 family members) expressed on exosomal surfaces.^[^ ^24^ ^]^ Encouraged by the crucial role of dendritic cells (DCs) in the antigen‐presentation procedure, a rational choice for optimal tumor antigenic stimulation may be to engineer a DC‐tumor chimeric exosome‐based nanovaccine.

In general, the therapeutic outcomes of antigen‐enriched cancer vaccines are enslaved to antigen‐presentation efficiency,^[^ ^25^, ^26^, ^27^ ^]^ and immunoadjuvants that potentiate the antigen‐presentation functionality are hence under intensive scrutiny. 2′3′‐cyclic guanosine monophosphate–adenosine monophosphate (cGAMP), a conventional stimulator of interferon genes (STING)‐activating cyclic dinucleotide (CDN) adjuvant, can exert a multifaceted inflammatory procedure to assist antigen‐presentation via the cytoplasmic pattern recognition receptor.^[^ ^28^, ^29^, ^30^, ^31^, ^32^ ^]^ While promising, the therapeutic efficacy of exogenous‐delivered cGAMP molecule is severely restricted by poor cytosolic entry due to its poor drug‐like properties.^[^ ^33^, ^34^ ^]^ cGAMP molecule with two phosphodiester bonds is an anionic, hydrophilic molecule, which is even susceptible to degradation by either intracellular or extracellular phosphodiesterase.^[^ ^35^, ^36^ ^]^

**Figure 1:**
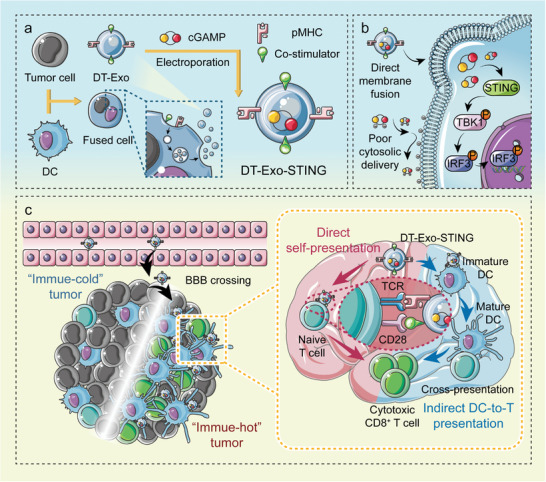
Schematic illustration of DT‐Exo‐STING nanovaccine for GBM immunotherapy. a) Design and fabrication of DT‐Exo‐STING nanovaccine. b) Schematic exhibiting action mode of this chimeric exosome‐assisted delivery strategy in STING activation. c) Mechanism of DT‐Exo‐STING–mediated antineoplastic immunoresponse in an intracranial GBM mouse model. P, phosphorylation; TBK1, TANK‐binding kinase 1; IRF3, interferon regulatory factor 3.

Ideally, developing an antigen‐enriched carrier loaded with CDN adjuvants would elicit a complementary immune response. To this end, we develop a DC‐tumor chimeric exosome‐based immunotherapeutic platform collaborated with co‐delivery of exogenous cGAMP molecular adjuvants (**Figure** **1**a). This exosome‐assisted delivery strategy rescues the potency of exogenous STING agonists (Figure 1b) to enhance antigen‐presentation performance within DCs and subsequent tumor‐specific T‐cell immunoresponse. In addition, DC‐tumor hybrid cells can impart their secreted nanovesicles with homologous characteristics to ensure the co‐loading of broad‐spectrum antigen‐derived pMHC and abundant costimulatory molecules. Thus, these chimeric nanovesicles can evoke a potent cytotoxic T‐cell response through direct self‐presentation avenue and indirect DC‐to‐T presentation manner, allowing for almost complete obliteration of intracranial glioblastoma multiforme (GBM) focuses (Figure 1c). Considering that tumor heterogeneity is deemed as a critical obstacle to clinical anti‐tumor immunotherapy, this therapeutic platform even exhibits a promising aptitude for personalized GBM therapy in a significant clinical setting of postoperative treatment.

## 2 Results and Discussion

### 2.1 Fabrication and Characterization of DT‐Exo‐STING

Inspired by the fusion‐driven integration of bi‐parental biological properties, together with the expertise of exosomes in therapeutic payload delivery,^[^ ^37^ ^]^ we proposed an immunotherapeutic nano‐platform using a DC‐tumor chimeric exosome‐based cancer vaccine for maximized tumor‐specific T‐cell response. In virtue of polyethylene glycol (PEG)‐induced fusion, DC‐tumor hybrid cells were obtained for further elaboration of nano‐sized vaccines.^[^ ^22^ ^]^ In the event of fusion initiation, the co‐loading of broad‐spectrum tumor antigen‐derived pMHC and co‐stimulatory molecules would occur in this cellular vaccine platform (Figure S1, Supporting Information).^[^ ^38^ ^]^ Fusion of the resulting DC‐tumor hybrid cells was identified through the double‐positive signaling of both classic tumor cell index (indicated by CD44) and DC indicator (marked by CD11c) (Figure S2, Supporting Information), subsequent to the execution of individual antibody staining toward GL261 cells and DCs.

**Figure 2:**
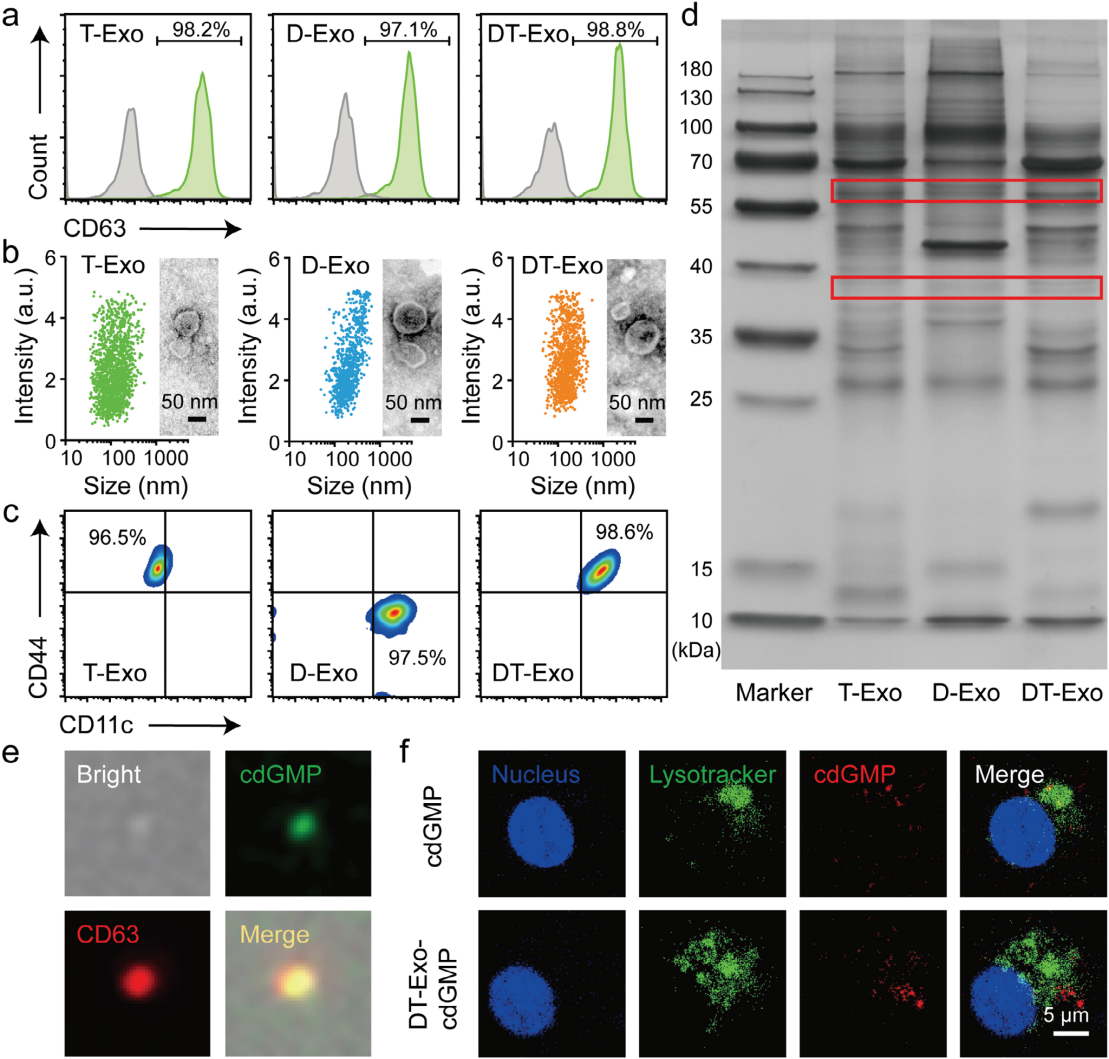
Preparation and characterization of DT‐Exo‐STING. a) Flow cytometry histograms of CD63 on T‐Exos, D‐Exos and DT‐Exos. b) TEM images and size distributions of T‐Exos, D‐Exos and DT‐Exos. c) Flow cytometry plots of anti‐CD44–labeled T‐Exos, anti‐CD11c–marked D‐Exos and the double‐antibody–labeled chimeic DT‐Exos. d) SDS‐PAGE protein analysis of T‐Exos, D‐Exos, and DT‐Exos. e) Imaging flow cytometry to determine the encapsulation of cdGMP‐Dy547 within DT‐Exos. cdGMP (green) and CD63 (red) in images. f) CLSM images of cdGMP‐Dy547 distribution within DCs after 4 h incubation with individual cdGMP‐Dy547 and cdGMP‐Dy547–loaded DT‐Exos. Nucleus (blue), lysosome (green), and cdGMP‐Dy547 (red) in confocal images.

Having confirmed the ideal hybridization of biparental cells, we proceeded to extract and purify both parental cell‐ and hybrid cell‐derived exosomes for further performance development. The determination of classical exosome protein CD63 on GL261 T‐Exos, DC‐derived exosomes (D‐Exos) and chimeric DC‐tumor exosomes (DT‐Exos) indicated that these exosomes were isolated with high‐purity (**Figure** **2**a; Figure S3, Supporting Information).^[^ ^39^ ^]^ Morphology assessment imparted additional proof to ascertain these obtained nanovesicles (Figure 2b; Figure S4, Supporting Information), through an emblematic cup‐shaped structure in transmission electron microscope (TEM) images and a size distribution trend of 40–160 nm in nanoparticle tracking analysis (NTA). A homologous signature of these nanovesicles inheriting from exosome‐secreting cells was confirmed (Figure 2c; Figure S5, Supporting Information) via the identical immunophenotypic results (Figure S2, Supporting Information). The fractions of T‐Exos, D‐Exos, and DT‐Exos were further subject to the sodium dodecyl sulfate‐polyacrylamide gel electrophoresis (SDS­‐PAGE) assay for protein expression analysis (Figure 2d). We noticed that the nearly entire protein expression of both T‐Exos and D‐Exos could be determined in DT‐Exos. Notably, several emerging protein bands belonging to chimeric nanovesicles were observed in red rectangles of Figure 2d, which might stand for the presence of certain biological functionalities of DT‐Exos distinguished from either T‐Exos or D‐Exos. Unlike the immunosuppressive T‐Exos, APC‐tumor chimeric exosomes were considered to express many immune‐activating microRNAs (miRNA).^[^ ^24^ ^]^ As exhibited in Figure S6a,b,c, Supporting Information, exosome *miR‐211‐3p* and *miR‐155‐5p* with immunostimulatory potential were up‐regulated within DT‐Exos versus T‐Exos, while immunosuppressive *miR‐187‐5p* in DT‐Exos was down‐regulated over T‐Exos.

Given the exosome‐intrinsic peculiarity in cell‐to‐cell communication,^[^ ^40^ ^]^ we proposed to conduct a versatile chimeric‐exosome–based carrier system for direct cytoplasmic delivery of intracellular‐acting immunomodulators. In the context of poor cytosolic entry, cGAMP molecules were the preferred payloads into this DT‐Exo nanovector platform for enhanced STING activation (DT‐Exo‐STING). Pursuing this, a fluorescently‐labeled CDN (cdGMP‐Dy547) was encapsulated into DT‐Exos (DT‐Exo‐cdGMP) using an electroporation technique. This encapsulation behavior was indeed achieved, with the observation of the colocalization of cdGMP‐Dy547 and exosomal indicator CD63 (Figure 2e). Note that no significant difference in encapsulation efficiencies of these CDN molecules emerged within both parental cell‐ and hybrid cell‐derived exosomes (Figure S7, Supporting Information). Given that exosome‐mediated intercellular communication hinges to a certain extent on the direct membrane fusion route,^[^ ^41^ ^]^ we next probed into whether this chimeric exosome could function as an ideal cargo vehicle to ameliorate the biological potency of interior drugs. As exhibited in the confocal laser scanning microscopy (CLSM) images (Figure 2f; Figure S8, Supporting Information), the fluorescence signal of DT‐Exo‐cdGMP was dispersed in the cytoplasm instead of colocalized with the lysosomes still 8 h, while cdGMP‐Dy547 was observed to be concentrated in the lysosomal compartments. This differentiated phenomenon indicated that DT‐Exos could achieve direct cytosolic entry of CDN‐based STING agonists, which was even comparable to the direct in vitro electroporation treatment (Figure S9, Supporting Information). We also observed that this chimera carrier‐assisted delivery strategy was conductive to the enhanced cell uptake of cdGMP‐Dy547 relative to free cargo molecules. In addition, this cellular uptake mechanism of membrane fusion was further confirmed through an inhibitor test (Figure S10, Supporting Information).

### 2.2 Dual T‐Cell Activation Elicited by Chimeric Exosome‐Based Nanovaccine

**Figure 3:**
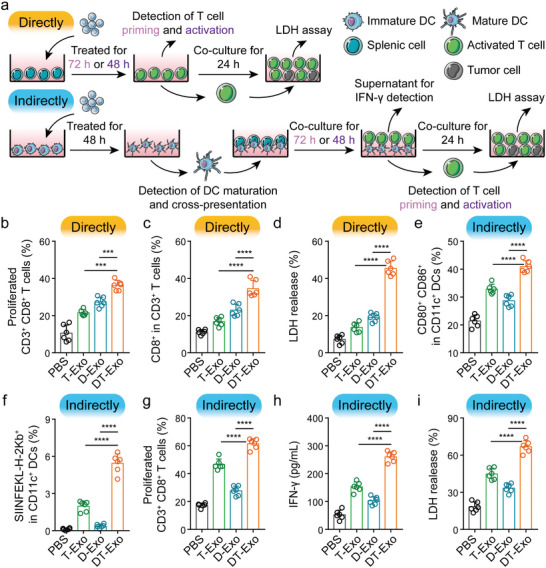
Dual activation of T cells by chimeric exosome‐based nanovaccine. a) Sketch depicting the dual T‐cell activation assays in (b to i). b) Flow cytometric quantification of splenic CD3^+^CD8^+^ T‐cell proliferation with the staining of CFSE after 72 h incubation directly with PBS, T‐Exos, D‐Exos, and DT‐Exos (*n* = 6; one‐way analysis of variance (ANOVA) with Tukey's multiple comparisons test). c) The proportions of CD3^+^CD8^+^ T cells 48 h period after treatment with the assigned formulations (*n* = 6; one‐way ANOVA with Tukey's multiple comparisons test). d) In vitro cytotoxicity of exosome‐activated T cells to GL261 cells 24 h after incubation with the designated formulations (*n* = 6; one‐way ANOVA with Tukey's multiple comparisons test). e) The quantitative evaluation of DC maturation (CD11c^+^CD80^+^CD86^+^) analyzed by means of flow cytometry post‐incubation with PBS, T‐Exos, D‐Exos, and DT‐Exos for 48 h (*n* = 6; one‐way ANOVA with Tukey's multiple comparisons test). f) Quantitative analysis of DC‐mediated cross‐presentation (CD11c^+^SIINFEKL‐H‐2Kb^+^) by flow cytometry, following 48 h incubation with the designated exosome nanovaccines (*n* = 6; one‐way ANOVA with Tukey's multiple comparisons test). g) The percentages of proliferated CD3^+^CD8^+^ T cells after 72 h co‐culture of CFSE‐stained splenic cells with the above‐pretreated DCs in a ratio of 20:1 (*n* = 6; one‐way ANOVA with Tukey's multiple comparisons test). h) IFN‐γ secretion in the supernatant of DC‐to‐T co‐culture system 48 h period post‐incubation of various exosome‐stimulated DCs and splenocytes with a ratio of 1:20, measured by ELISA kit (*n* = 6; one‐way ANOVA with Tukey's multiple comparisons test). i) In vitro cytotoxicity mediated by the designated exosome‐DC–treated splenocytes to GL261 cells 24 h post‐incubation with an effector/target ratio of 10:1 (*n* = 6; one‐way ANOVA with Tukey's multiple comparisons test). Data in (b to i) are represented as means ± SD. ***p < 0.001, ****p < 0.0001.

In the case of DC‐tumor fusion initiation, hybrid cells would be equipped with broad‐spectrum pMHC derived from the processed whole tumor antigens and plentiful co‐stimulatory molecules.^[^ ^38^ ^]^ In this context, their secreted exosomes can inherit the desired versatile functionalities.^[^ ^24^ ^]^ Resemble to the immunostimulatory properties of intact DCs, these chimeric nanovesicles can directly present tumor antigens to T lymphocytes and then signal both proliferation and activation into these cells. Like tumor cells, the immunogenic antigen‐containing DT‐Exos are confronted with the recognition and uptake by DCs, prior to antigen presentation to T cells for the priming of tumoricidal immune response. These aforementioned promising avenues for both direct and indirect optimization of T‐cell functionality were assessed as illustrated in **Figure** **3**a. Upon the cooperation of tumor antigen‐derived pMHC (Figure S11, Supporting Information) and co‐stimulatory molecules on DT‐Exos, similar significant rising trends were observed in both proliferative response of carboxyfluorescein succinimidyl ester (CFSE)‐stained splenocytes (Figure 3b; Figure S12a, Supporting Information) and activation signaling of CD3^+^CD8^+^ cytotoxic T lymphocytes (Figure 3c; Figure S12b, Supporting Information). To further evaluate the tumoricidal properties of these T lymphocytes, the exosome‐activated splenocytes were subject to lactate dehydrogenase (LDH) release assay upon 24 h co‐incubation with GL261 cells (Figure 3d). The splenocytes treated with DT‐Exos exhibited an aggravated virulence toward GL261 cells versus both T‐Exos and D‐Exos. Nevertheless, treatment with either T‐Exos or D‐Exos merely elicited a moderate increase in these proposed T‐cell functionalities, which was credited to a simultaneous lack of effective antigen presentation and abundant costimulatory molecule expression.^[^ ^42^ ^]^ These phenomena shed light on the feasibility that such chimeric exosome‐based nanovaccines could act as nano‐sized APCs and replace intact counterparts to trigger a direct immune‐activated response on cytotoxic CD8^+^ T lymphocytes.

As regards DT‐Exo‐mediated indirect immune activation, one might notice that DCs could exert a significant impact on the inflammatory response, especially in the context of bridging T‐cell‐activated immunomodulatory pathway.^[^ ^43^ ^]^ This circumstance invigorated a fervor of comparative exploration into various exosome‐stimulated mouse bone marrow‐derived dendritic cell (BMDC) properties. In theory, the occurrence of DC maturity would render an intensified furnishment with costimulatory factors CD80 and CD86 on cellular surface. Along with this guideline, BMDCs were subject to 48‐hour co‐incubation with exosome formulations for further immunophenotypic analysis. We noticed a dramatic increment of mature signals in BMDCs stimulated with DT‐Exos relative to either T‐Exos or D‐Exos. Whereas, a suboptimal immunostimulatory response was observed in T‐Exo–treated BMDCs despite both T‐Exos and DT‐Exos containing immunogenic antigen moiety (Figure 3e; Figure S13a, Supporting Information). A postulated merit of DT‐Exos in actuating DC maturation might be to possess the pre‐fabricated pMHC derived from broad‐spectrum tumor antigen, guaranteeing the premium quality of antigen presentation.^[^ ^22^ ^]^ To further certify this, ovalbumin (OVA)‐expressing GL261 cell line (GL261‐OVA) was introduced into the manufacturing process of these chimeric nanovesicles. Note that a significant enhancement of DC‐mediated cross‐presentation signal (CD11c^+^SIINFEKL‐H‐2Kb^+^) in DT‐Exo‐stimulated BMDCs occurred (Figure 3f; Figure S13b, Supporting Information). In principle, these two avenues of DC performance optimization would contribute to the subsequent priming of cytotoxic CD8^+^ T lymphocyte‐mediated anti‐tumor response, especially in T‐cell proliferation and tumoricidal potentiality. As expected, this indirect DC‐to‐T treatment with DT‐Exos resulted in a remarkable increment of proliferative signaling in CD3^+^CD8^+^ splenocytes (Figure 3g; Figure S14, Supporting Information). This phenomenon indicated that such a DT‐Exo‐induced indirect immune activation conferred CD8^+^ cytotoxic T lymphocytes with access to optimal antigen recognition, consistent with the tendency of DC‐mediated antigen cross‐presentation. Trend similarity of proinflammatory cytokine interferon‐γ (IFN‐γ) secretion, a predictive indicator for T‐cell tumoricidal potentiality, was also observed in DT‐Exo‐DC–treated splenocytes under the detection of enzyme‐linked immunosorbent assay (ELISA) in Figure 3h. To further intuitively determine this T lymphocyte‐mediated antitumor cytotoxicity, LDH release assay was performed 24 h post‐incubation of DT‐Exo‐DC–activated splenic T cells and GL261 cells (Figure 3i). This indirect DC‐to‐T treatment with DT‐Exos exerted enhanced toxicity toward GL261 cells over both T‐Exos and D‐Exos. All these observations demonstrated that this classic DC‐to‐T immunostimulatory route activated by DT‐Exos resulted in the functional improvement of T lymphocytes.

Encouraged by the observed scenario in Figure 2f, a rational hypothesis was proposed that the direct cytosolic delivery of CDN adjuvants mediated by DT‐Exos would contribute to STING activation within DCs. To this end, DT‐Exo‐STING–stimulated BMDCs were subject to quantitative flow cytometric evaluation of phosphorylated interferon regulatory factor 3 (p‐IRF3), which was a mobilizer for downstream signaling cascades and transcriptional activation.^[^ ^44^ ^]^ Indeed, such chimeric exosome‐assisted delivery strategy rescued cGAMP activity as reflected in the dramatic augment of p‐IRF3 response, while a delicate activation degree of this immune sensor was noted in free cGAMP‐stimulated BMDCs even comparable to phosphate‐buffered saline (PBS) treatment (Figure S15a,b, Supporting Information). We were astonished that an individual DT‐Exo treatment also incurred an approximately 3‐fold enhancement of p‐IRF3 expression above the baseline. This phenomenon supported the documented exosome‐coordinated immunomodulation, that is, exosomal DNA‐induced STING activation within recipient cells.^[^ ^45^ ^]^ In addition, the stability of this exosome‐based nanovector within a 2‐month period was evaluated through size characterization in Figure S16a (Supporting Information), and the potency of STING agonists within DT‐Exo‐STING was also observed to be stable during preservation in Figure S16b (Supporting Information). Together, DT‐Exos could potentiate T‐cell functionality in both proliferative response and tumoricidal capability and enhance the biological potency of intracellular‐acting immunomodulators.

### 2.3 Tissue Tropism of DT‐Exo‐STING in Vivo

**Figure 4:**
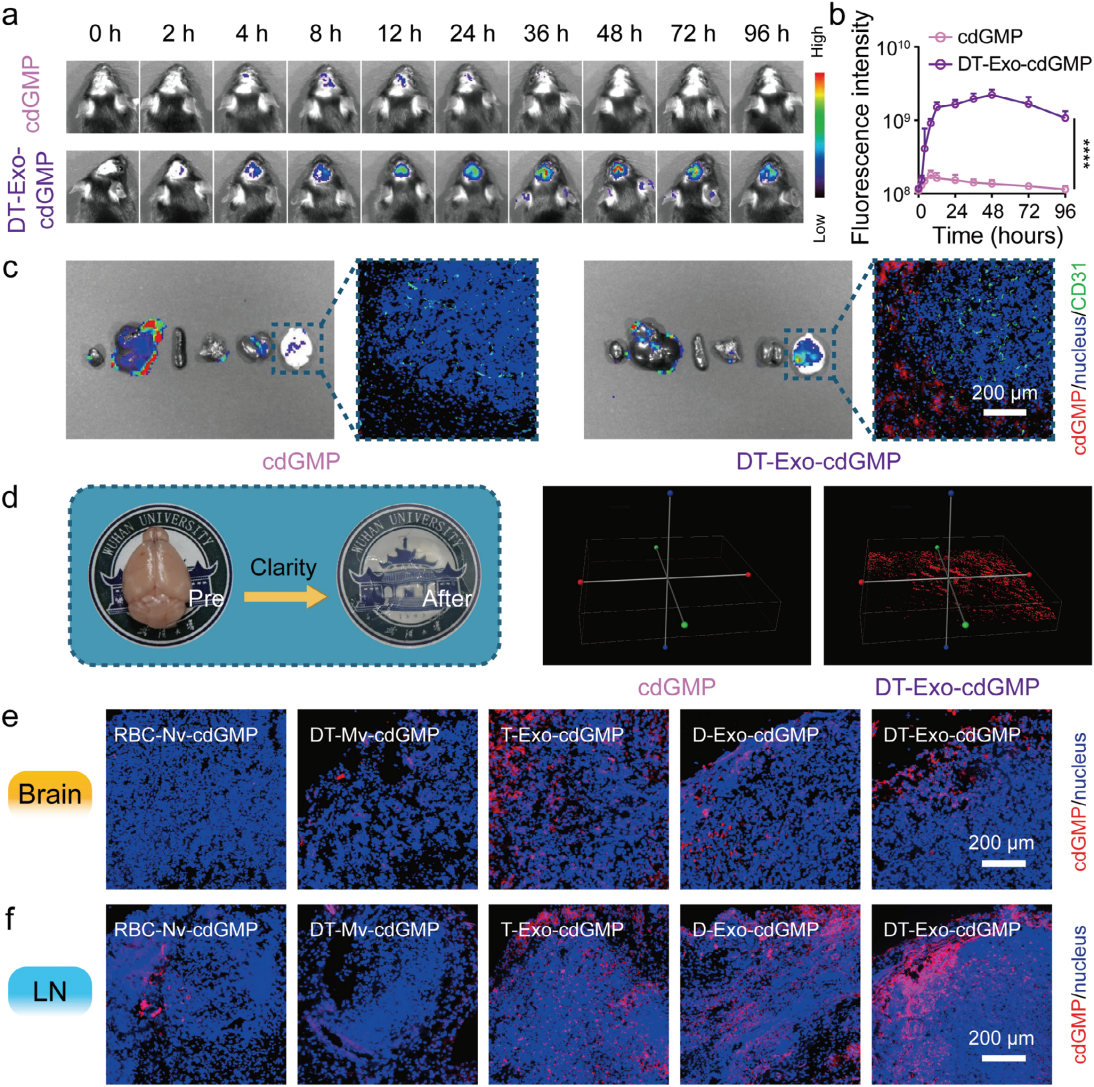
Targeted delivery of DT‐Exo‐STING in vivo. a) Representative in vivo fluorescence imaging and b) quantification in brain regions of GL261‐bearing mice at the indicated time points after subcutaneous administration with cdGMP‐Dy547–containing formulations (*n* = 3; two‐way ANOVA with Tukey's multiple comparisons test). c) Ex vivo fluorescence imaging of the major organs (heart, liver, spleen, lung, kidney, and brain) 12 h after subcutaneous administration. Inserted images exhibited the brain penetration of cdGMP‐Dy547–containing formulations. cdGMP‐Dy547 (red), nucleus (blue), and CD31 (green) in images. d) 3D fluorescence imaging to visualize the distribution of cdGMP‐Dy547–loaded DT‐Exos within the optically transparent brain tissues performed with CLARITY technique. cdGMP‐Dy547 (red) in images. e) Ex vivo fluorescent images of brain tissues and f) cervical LNs of GL261 tumor‐bearing mice following subcutaneous injection with various vesicles containing cdGMP‐Dy547. cdGMP‐Dy547 (red) and nucleus (blue) in images. Data in (b) are represented as means ± SD. ****p < 0.0001.

Exosomes are deemed as intercellular couriers for either short‐haul or systemic signal communications, during which these nanovesicles can carry therapeutic payloads across certain intractable anatomic barriers.^[^ ^37^ ^]^ In this context, we proposed a rational inference that such chimeric drug‐loaded nanodevices might reconcile targeting demands of BBB‐crossing delivery. To investigate whether the utility of DT‐Exos as the vectors could accomplish the targeted delivery of exogenous cGAMP molecules to intracranial tumors, these cdGMP‐Dy547–containing formulations were subcutaneously injected into the back of GL261‐burdened mice. A DT‐Exo‐cdGMP treatment led to a progressive accumulation of fluorescence signal in the brain regions still 48 h and residual signal could be discoverable at 96 h, while free cdGMP‐Dy547 molecules were virtually undetectable above the background (**Figure** **4**a,b). A similar situation also emerged in the healthy mice (Figure S17, Supporting Information). The brain‐tropism behavior of this chimeric nanocarrier was again confirmed through the biodistribution within the major organs (heart, liver, spleen, lung, kidney, and brain) (Figure 4c; Figures S18 and S19, Supporting Information). The noncoincident fluorescence signal of the endothelial cells (marked by CD31) and cdGMP‐Dy547 further indicated that DT‐Exo‐cdGMP could enter the brain parenchymal (Figure 4c). In addition, the moderate accumulation of DT‐Exo‐cdGMP was observed within liver tissues second only to brains, while there was no significant change in other organs. To assess the possible immune toxicity of the treatments, we next evaluated the pro‐inflammatory cytokines including monocyte chemoattractant protein 1 (MCP‐1), interleukin 6 (IL‐6), and tumor necrosis factor α (TNF‐α) of liver tissues 4 days after administration (Figure S20a,b,c, Supporting Information). DT‐Exo‐cdGMP treatment was observed with no obvious immunotoxicity to mouse livers. Utilizing the surfactant assistant tissue clearing technology, so‐called CLARITY,^[^ ^46^ ^]^ and three‐dimensional (3D) fluorescence imaging techniques, we found a noteworthy aggregation of fluorescence signal within the intracranial targeted regions of DT‐Exo‐cdGMP–treated mice (Figure 4d), reconfirming the distinguished BBB‐crossing capability of this chimeric exosome‐based nanocarrier.

Given this observed brain‐tropism phenomenon, we proceeded to determine the elements interrelated with BBB‐crossing property. According to the documented studies, the major objects accountable to the targeting capacity of exosome‐based vectors were nominated as the size and constituent of these nanovesicles.^[^ ^47^ ^]^ Pursuing this, a succession of cdGMP‐Dy547–loaded cellular vesicles, consisting of red blood cell‐derived nanovesicles (RBC‐Nv‐cdGMP), chimeric DC‐tumor micro‐sized vesicles (DT‐Mv‐cdGMP) and three proposed exosomes (T‐Exo‐cdGMP, D‐Exo‐cdGMP and DT‐Exo‐cdGMP), was prefabricated for further oriented observation. Nanoparticles with a diameter distribution of 50–200 nm, in general, are inclined to cross the BBB, and thus accumulate in intracranial regions.^[^ ^48^ ^]^ Along this perspective, a significant enhancement of fluorescence signal intensity was observed within brain tissues post‐administration of all three cdGMP‐Dy547–loaded exosomes, relative to large‐sized DT‐Mv‐cdGMP with the faint enrichment level (Figure 4e). Of note, the identical nano‐sized RBC‐Nv‐cdGMP did not evoke a distinct amassment of fluorescence signal within brain regions even inferior to DT‐Mv‐cdGMP. This differentiated phenomenon manifested that an active‐targeting avenue driven by superficial components of these vesicles presumably dominated the brain‐tropic migration instead of size‐mediated passive targeting. Inspired by the observation that enhanced accumulation of both T‐Exo‐cdGMP and DT‐Exo‐cdGMP versus D‐Exo‐cdGMP emerged within brain tissues, we surmised that the ascendant BBB‐crossing manifestation of this chimera carrier might be, to a large extent, credited to tumor‐homing property inherited from parental hybrid cells.^[^ ^49^ ^]^

Having noticed this brain‐tropism behavior, we then attempted to explore whether these chimeric exosomes acquired in like manner the DC‐specific targeting signature from parental hybrid cells, that is, lymph node (LN)‐homing capacity (Figure 4f). Either D‐Exo‐cdGMP or DT‐Exo‐cdGMP elicited a noteworthy increment of LN‐targeting tendency exceeding T‐Exo‐cdGMP, substantiating the LN‐draining performance actuated by DC‐related moieties within this chimeric exosome‐based drug carrier,^[^ ^50^ ^]^ wherein optimum LN tropism was observed in DT‐Exo‐cdGMP–treated mice. The disparity between D‐Exo– and DT‐Exo–driven LN tropism might be concerned with the equipment of LN‐homing molecules on their surfaces. Unlike immature DCs, DC‐tumor hybrid cells are subject to antigenic stimulus in the fusion procedure, and then furnished with immense amounts of LN‐homing molecules on their cytomembranes.^[^ ^22^ ^]^ After that, their shed chimeric exosomes can inherit homologous functionalities with intensified LN‐homing capacity. Overall, these immunomodulator‐loaded nanocarriers were up to the mustard of dual‐tropism delivery into both LN regions and malignant lesions, even across the BBB, for further reinvigoration of antineoplastic immune response.

### 2.4 DT‐Exo‐STING–Enhanced Therapeutic Efficacy in an Orthotopic GBM Model

**Figure 5:**
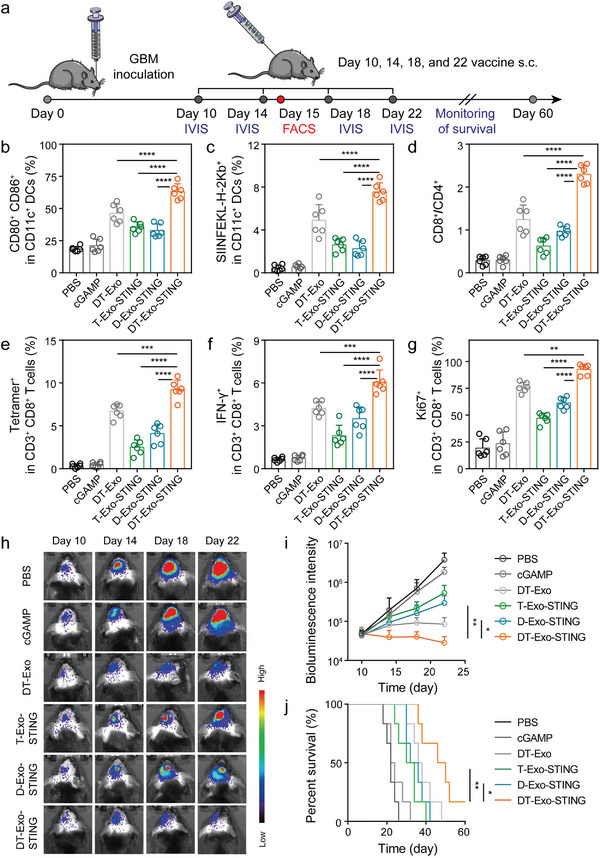
DT‐Exo‐STING–induced antineoplastic efficacy in an orthotopic GBM mouse model. a) Treatment scheme for mice with intracranial GL261‐OVA‐Luc tumors. Mice were subject to subcutaneous administration with cGAMP‐containing formulations a total of four times, 4 days apart. b) Flow cytometric quantification of mature DCs (CD11c^+^CD80^+^CD86^+^) in cervical LNs of mice treated with the designated vaccine formulations (*n* = 6; one‐way ANOVA with Tukey's multiple comparisons test). c) The proportions of CD11c^+^SIINFEKL‐H‐2Kb^+^ DCs in cervical LN tissues analyzed by means of flow cytometry (*n* = 6; one‐way ANOVA with Tukey's multiple comparisons test). d) Ratio of tumor‐infiltrating CD8^+^ to CD4^+^ T cells (gated from CD3^+^ cells; *n* = 6; one‐way ANOVA with Tukey's multiple comparisons test). e) Quantitative analysis of H‐2Kb/SIINFEKL tetramer staining of CD3^+^CD8^+^ T cells within tumors, using flow cytometry (*n* = 6; one‐way ANOVA with Tukey's multiple comparisons test). f) The percentages of IFN‐γ^+^ T cells in tumor tissues (gated from CD3^+^CD8^+^ cells; *n* = 6; one‐way ANOVA with Tukey's multiple comparisons test). g) The quantification of Ki67 expression in tumor‐infiltrating CD3^+^CD8^+^ T cells (*n* = 6; one‐way ANOVA with Tukey's multiple comparisons test). h) Representative in vivo bioluminescence images and i) quantified signal intensity of glioma‐bearing mice after the indicated treatments (*n* = 3; two‐way ANOVA with Tukey's multiple comparisons test). j) Kaplan–Meier survival curves of mice vaccinated with the designated cancer vaccine formulations (*n* = 6; log‐rank Mantel–Cox test). Data in (b to g, and i) are represented as means ± SD. *p < 0.05, **p < 0.01, ***p < 0.001, ****p < 0.0001. s.c., subcutaneous.

Encouraged by the aforementioned results, we further investigated whether DT‐Exo‐STING could exert a potent and durable immune response in vivo utilizing a poorly immunogenic GL261 syngeneic orthotopic GBM model.^[^ ^51^ ^]^ To assess in vivo cellular uptake property of CDN adjuvants delivered by this chimeric nanocarrier, GBM‐bearing mice were subcutaneously injected with DT‐Exo‐cdGMP. DT‐Exo‐cdGMP was observed to be most commonly localized within DCs (CD11c^+^) 12 h after administration (Figure S21, Supporting Information). On day 10 post‐inoculation of luciferase‐expressing GL261‐OVA (GL261‐OVA‐Luc) glioma cells, mice were subject to subcutaneous administration with PBS solution, free cGAMP, individual DT‐Exos, STING‐activating cGAMP‐loaded T‐Exos (T‐Exo‐STING), STING‐activating cGAMP‐containing D‐Exos (D‐Exo‐STING) and DT‐Exo‐STING as depicted in **Figure** **5**a. Mice were euthanized 24 h after two injection rounds, and corresponding tissues were harvested for further flow cytometric analysis. DT‐Exo‐STING treatment dramatically elicited an elevated level of DC maturation (CD11c^+^CD80^+^CD86^+^) inside the cervical LNs of treated mice relative to vaccination with either T‐Exo‐STING or D‐Exo‐STING, while free cGAMP did not exert an immunostimulatory response of DCs above the baseline (Figure 5b; Figure S22a, Supporting Information). We also uncovered that optimal performance of DC‐mediated antigen cross‐presentation (CD11c^+^SIINFEKL‐H‐2Kb^+^) within the cervical LNs was achieved by cGAMP‐loaded DT‐Exos superior to bare DT‐Exos, as a forthright proof that exosome‐assisted CDN delivery strategy could further amplify antigen‐presentation properties of DCs (Figure 5c; Figure S22b, Supporting Information).

Having noted the immune‐activated DCs, we proceeded to probe into the antitumor T‐cell response within intracranial tumor tissues. Treatment with both individual DT‐Exos and DT‐Exo‐STING exhibited an obvious reversal in the ratio of tumor‐infiltrating CD3^+^CD8^+^ T lymphocytes to CD3^+^CD4^+^ T lymphocytes (CD3^+^CD8^+^/CD3^+^CD4^+^ > 1), a generally reported prognostic metric relevant to clinical immune efficacy (Figure 5d; Figure S23a, Supporting Information). More specifically, DT‐Exo‐STING treatment elicited a significant boost in the amount of intratumoral antigen‐specific T cells, as noted by the increases of CD3^+^CD8^+^SIINFEKL‐MHC‐I tetramer^+^– and CD3^+^CD8^+^IFN‐γ^+^–stained lymphocytes (Figure 5e,f; Figure S23b,c, Supporting Information). In addition, we also noticed an elevated Ki67 response to DT‐Exo‐STING nanovaccine in CD3^+^CD8^+^ T cells within intracranial tumor regions (Figure 5g; Figure S23d, Supporting Information). Significantly, a positive modulatory role of D‐Exo‐STING was observed in the potent T‐cell functionality of differentiation property, tumoricidal capacity, and proliferative potential versus T‐Exo‐STING. Whereas, such moderate optimization of D‐Exo‐STING–induced immunoinflammatory response in DCs did not emerge again. This circumstance might be implicated in the immune escape mechanism of T‐Exos, such as program death‐ligand 1 (PD‐L1)‐mediated T‐cell dysfunction.^[^ ^52^ ^]^ Furthermore, DT‐Exo‐STING treatment significantly increased the proportion of the central memory T cells (T_CM_; CD8^+^CD44^+^CD62L^+^) in the peripheral blood of mice 24 h after injection cessation (Figure S23e,f, Supporting Information). This phenomenon indicated that such a DT‐Exo‐STING nanovaccine could enhance the anti‐tumor functionality of CD8^+^ cytotoxic T lymphocytes in long‐term immunological memory response. In addition, the immune‐activating miRNAs including *miR‐211‐3p* and *miR‐155‐5p* were up‐regulated within the tumor tissues of mice treated with DT‐Exos versus PBS group, while the down‐regulated trend of immunosuppressive *miR‐187‐5p* was observed in the tumors of DT‐Exo–treated mice (Figure S24, Supporting Information).

Profiting from the chimeric exosome‐driven inflammatory program, subcutaneous administration with DT‐Exo‐STING yielded a substantial treatment benefit in both the insensible change of GBM tumor growth rate and significant prolong in the survival time over either T‐Exo‐STING or D‐Exo‐STING (median survival (MS)_T‐Exo‐STING_ = 32 days; MS_D‐Exo‐STING_ = 34 days; MS_DT‐Exo‐STING_ = 48 days) in Figure 5h–j. A supportive role of cGAMP adjuvants within chimeric exosomes was also observed with a notable therapeutic benefit over bare DT‐Exos, whereas improved treatment outcomes were not monitored in free cGAMP treatment even comparable to the baseline. In addition, a subtle balance between immunostimulatory activity and biosafety was a prerequisite for well‐developed clinical manifestation. In this context, hematoxylin and eosin (H&E) slide examinations were performed with the observation of negligible damage to major organs by these exosome‐containing formulations (Figure S25, Supporting Information).

### 2.5 Personalized DT‐Exo‐STING Combined with ICB Therapy Prevented Post‐Operative GBM Recurrence

**Figure 6:**
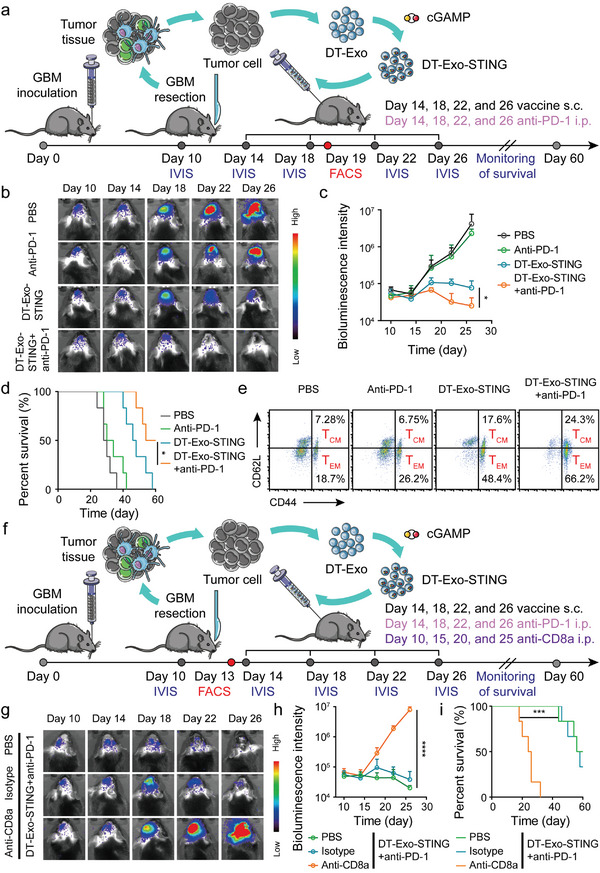
Combinatorial efficacy of personalized DT‐Exo‐STING and ICB therapy against postsurgical GBM recurrence. a) Postoperative treatment scheme for GL261‐Luc–burdened mice. Mice were treated subcutaneously with personalized DT‐Exo‐STING and intraperitoneally with anti‐PD‐1 antibodies (ICB) a total of four times, 4 days apart, subsequent to intracranial GBM surgical resection. b) In vivo fluorescence imaging and c) quantification of GL261‐Luc–bearing mice treated with the indicated formulations (*n* = 3; two‐way ANOVA with Tukey's multiple comparisons test). d) Kaplan–Meier survival analysis of mice after various treatments (*n* = 6; log‐rank Mantel–Cox test). e) Representative flow dot plots of T_EM_ (CD44^+^CD62L^−^; gated from CD8^+^ cells) and T_CM_ (CD44^+^CD62L^+^; gated from CD8^+^ cells) in the peripheral blood on day 19 post‐inoculation of GBM tumors. f) Schematic illustration of the experimental design combining chimeric exosome‐based nanovaccine and ICB therapy against post‐operative GBM recrudescence. Mice were treated subcutaneously with personalized DT‐Exo‐STING and intraperitoneally with ICB therapy a total of four times, 4 days apart, and received intraperitoneal injections with anti‐CD8a or isotype monoclonal antibody IgG total four times, 5 days apart. g) Representative in vivo bioluminescence images and h) quantification of bioluminescence signal strength in GL261‐Luc–burdened mice pre‐treated with anti‐CD8a or mouse monoclonal IgG, prior to the combination treatment of chimeric exosome‐based nanovaccine and ICB therapy (*n* = 3; two‐way ANOVA with Tukey's multiple comparisons test). i) Kaplan–Meier survival curves of mice immunized with the combined therapy and other assigned formulations (*n* = 6; log‐rank Mantel–Cox test). Data in (c and h) are represented as means ± SD. *p < 0.05, ***p < 0.001, ****p < 0.0001. i.p., intraperitoneal.

Surgical debulking is the preferred clinical therapy feasible for patients with GBM tumors, especially in the setting of advanced tumors. In this context, methods that harness autologous‐derived tumor tissues for customized patient‐specific cancer vaccines have long been a priority in the immunotherapy field. We assessed the therapeutic efficacy of post‐operative treatment options with tight clinical correlation using personalized DT‐Exo‐STING nanovaccine. To this end, surgical excision of the visible glioma was executed 10‐day period after inoculation of mice with luciferase‐expressing GL261 (GL261‐Luc) cells. The resected GBM tumor tissues were further elaborated for autologous tumor‐moiety–containing DT‐Exo‐STING nanovaccine, prior to collaboration with immune checkpoint blockade (ICB) therapy (**Figure** **6**a). Combining subcutaneous DT‐Exo‐STING treatment with intraperitoneal administration of anti‐programmed death 1 (anti‐PD‐1) exerted almost complete eradication of recurrent GBM focuses, and even an individual DT‐Exo‐STING treatment could distinctly postpone the recurrence progression of residual lesions (Figure 6b,c). Improved responses to ICB therapy were also observed in Figure 6d through the significant survival benefits of mice vaccinated with DT‐Exo‐STING plus anti‐PD‐1 therapy versus individual DT‐Exo‐STING (MS_DT‐Exo‐STING_ = 47 days; MS_DT‐Exo‐STING + anti‐PD‐1_ = 57 days).

Having noted the DT‐Exo‐STING–mediated sensitivity to ICB therapy, we then attempted to explore whether this personalized nanovaccine would impart a robust and durable memory T‐cell immunity as a potent collaborator with ICB therapy. Of note, a synchronous increasing trend occurred for effector memory T cells (T_EM_; CD8^+^CD44^+^CD62L^−^) and T_CM_ (CD8^+^CD44^+^CD62L^+^) in the peripheral blood of mice with DT‐Exo‐STING treatment in combination with ICB therapy (Figure 6e; Figure S26a,b, Supporting Information). This phenomenon manifested that the combinatorial strategy potentiated the CD8^+^ T‐lymphocyte functionality in both transient and long‐term immunological memory response. Given the vital importance of cytotoxic CD8^+^ T cells in ICB response, an additional experiment connected with T‐cell blocking was executed to substantiate the CD8^+^ T‐cell dependency for this combinatorial immunotherapeutic strategy (Figure 6f). A virtually entire blockade of CD8^+^ T cells by CD8a monoclonal antibody (anti‐CD8a) in the peripheral blood was distinctly observed, while treatment with immunoglobulin G (IgG) as control elicited a negligible change for CD8^+^ T cells (Figure S27, Supporting Information). As anticipated, CD8^+^ T‐cell blockade substantially hampered the therapeutic outcomes of DT‐Exo‐STING treatment plus ICB therapy, which resulted in only a trivial suppression of neoplasm recurrence (Figure 6g,h) that did not confer a significant survival benefit (MS_PBS + DT‐Exo‐STING + anti‐PD‐1_ = 58 days; MS_Anti‐CD8a + DT‐Exo‐STING + anti‐PD‐1_ = 25 days) (Figure 6i).

## 3 Conclusion

Leveraging fusion‐guided integration of biparental biological properties, we have engineered a versatile DC‐tumor chimeric exosome‐based nano‐platform, functionalized with CDN adjuvant‐driven STING activation. This chimeric exosome‐assisted delivery tactic can exert multifaceted merits, even superior to off‐the‐shelf CDN delivery techniques on matters of 1) the desirable cytosolic delivery of STING agonists into APCs, owing to exosome‐intrinsic property of cell‐to‐cell communication; 2) the anticipated optimization of CD8^+^ T‐cell–mediated immunostimulatory response through both direct and indirect immuno‐activation pathways, particularly in proliferation potency and tumoricidal activity; and 3) the brilliant tissue‐homing capacity consistent with exosome‐secreting DC‐tumor hybrid cells, even across intractable BBB. It is noteworthy that, the upscaling approach through elaborate fabrication of resected tumor tissues into this personalized antigen‐presentation platform, to a certain extent, guarantees the feasibility of revitalizing autologous tumor antigen‐reactive T cells. As a monotherapy, local administration of this DT‐Exo‐STING nanovaccine can yield a systemic and complete immune response, even adequate to virtually obliterate either intracranial primary or postoperative leftover tumor focuses. In view of this personalized vaccine strategy, such a chimeric exosome‐based therapeutic platform also can improve responses to ICB immunotherapy, rendering an ever‐expanding set of possibilities in clinical exploratory development. While not investigated herein, one may expect that such a DT‐Exo‐based delivery platform would allow for the encapsulation of multiple therapeutic payloads, imparting prospective options for the co‐delivery of STING agonists with other intracellular‐functioning therapeutics. Beyond that, this modularized system, as a class of cell‐free vaccines, also possesses an unparalleled advantage distinguished from conventional adoptive cell therapy, that is, long‐term storage shelf life. Thus, the versatility of this chimeric exosome‐based delivery platform imparts application prospects in the clinical realm, as a potent toolkit to unleash the potentiality of an autologous immune system.

## 4 Experimental Section

### Animals and Cell Lines

All animal experimentations were approved by the Institutional Animal Care and Use Committee (IACUC) of the Animal Experiment Center of Wuhan University (Wuhan, China) under protocol WP20210488. All animal experimental procedures were executed abiding by the Regulations for the Administration of Affairs Concerning Experimental Animals approved by the State Council of the People's Republic of China.

GL261 and GL261‐Luc murine glioblastoma cells were cultured in Dulbecco's Modified Eagle Medium (DMEM; Invitrogen) supplemented with 10% fetal bovine serum (FBS; Biological Industries) and 1% streptomycin/penicillin (Biological Industries). For the acquisition of a stabilized GL261‐Luc cell line, 10 µg mL^−1^ puromycin (Beyotime) was added into the media. BMDCs were derived from induced differentiation of bone mesenchymal stem cells (BMSCs).^[^ ^53^ ^]^ In brief, both femurs and tibias of female C57BL/6 mice (6–8 weeks old) were collected, and these bone joint heads were removed to expose the bone marrow cavities. Thereafter, BMSCs were flushed from mouse marrow cavities and resuspended in the RPMI‐1640 medium (Invitrogen) containing 10% heat‐inactivated FBS, 1% penicillin/streptomycin, 20 ng mL^−1^ recombinant granulocyte‐macrophage colony‐stimulating factor (GM‐CSF; Beyotime) and 10 ng mL^−1^ recombinant interleukin‐4 (IL‐4; Beyotime). After a 6‐day induced differentiation, BMDCs were harvested for further use. All cells were maintained at 37 °C with 5% CO_2_ in a humidified atmosphere.

### Fusion of BMDCs and Tumor Cells

The cell fusion of BMDCs and tumor cells was performed with reference to PEG (Sinopharm Chemical Reagent)‐fusion protocol.^[^ ^22^ ^]^ Briefly, well‐differentiated BMDCs and tumor cells pretreated with 20% (v/v) alcohol (Sinopharm Chemical Reagent) for cell inactivation were mixed at a ratio of 2:1 and centrifuged at 500 g for 10 min. Then, 1 mL pre‐configured solution containing 50% PEG (MW: 4000; w/w) and 10% (v/v) dimethyl sulfoxide (DMSO; Sinopharm Chemical Reagent) was added dropwise into the centrifuged precipitate prior to the homogeneous dispersion of cells. After a 2‐min incubation at 38 °C, the cell fusion was terminated through the drip addition of serum‐free RPMI 1640 medium. The well‐fused cells were further purified by centrifugation at 500 g to remove the excess PEG and resuspended in RPMI‐1640 medium containing 10% FBS, 1% penicillin/streptomycin, and 10 ng mL^−1^ IL‐4.

### Preparation and Characterization of DT‐Exos

Bare GL261 T‐Exos, D‐Exos, and chimeric DT‐Exos were separated and purified through serial centrifugation method. These exosome‐secreting cells were cultivated in an exosome‐depleted medium, wherein FBS was preprocessed by 3 h ultracentrifugation at 200 000 g. The collected supernatants were centrifuged at 300 g for 10 min and then 2000 g for 10 min, prior to 30‐min centrifugation at 10 000 g for the removal of cell debris. Afterward, the filtrate underwent ultracentrifugation at 100 000 g for 2 h, and the obtained exosomes were resuspended in a PBS buffer. Exosomes were stored at −80 °C or used freshly. For detection of exosome extraction, these exosome samples were stained with fluorochrome‐labeled antibody against canonical exosome protein CD63 (BioLegend; NVG‐2) for 30 min at 4 °C before flow cytometric analysis. The morphological characteristics and size distribution of exosomes were evaluated using TEM (Hitachi HT7700) and NTA (NanoSight LM10) respectively. The presence of chimeric exosomes was confirmed by the identification of classical indicators. In brief, GL261 cells were stained with anti‐CD44 (BioLegend; IM7), and DCs were marked with anti‐CD11c (BioLegend; N418), prior to cell fusion initiation. Exosomes shed by these antibody‐labeled cells were subject to flow cytometric analysis. All flow cytometric data were acquired on flow cytometry (BD Accurit C6 Plus) and analyzed utilizing FlowJo software.

### Preparation and Characterization of DT‐Exo‐STING

cGAMP (InvivoGen) molecules were loaded into exosomes utilizing a Gene Pulser Xcell electroporation system (Bio‐Rad). A total of 100 µL prepared electroporation mixture comprising exosomes (100 µg mL^−1^) and cGAMP molecules (100 µg mL^−1^) was suspended in the ice‐cold 4‐mm electroporation cuvette, and then electroporation was operated with a parameter setting of 200 V. After washed with PBS buffer, the purified DT‐Exo‐STING nanovaccines were ultimately obtained. This embarkation behavior was assessed using a fluorescently‐labeled CDN, that is, cdGMP‐Dy547 (Axxora) under the surveillance of an imaging flow cytometer (Amnis ImageStreamX Mk II), and the encapsulation efficiencies into diverse exosomes were further determined by LS55 fluorescence spectrometer (Perkin‐Elmer). To evaluate exosome‐mediated direct cytosolic delivery of CDN adjuvants, well‐differentiated BMDCs were seeded in a glass‐bottomed cultured dish with a density of 500 000 cells well^−1^ and subject to 4 h incubation with cdGMP‐Dy547 alone and cdGMP‐Dy547–containing DT‐Exos at dosage of cdGMP‐Dy547 (100 ng mL^−1^). After staining with Hoechst 33342 and Lysotracker green, cells were observed with a CLSM (Leica Microsystems).

### In Vitro Dual T‐Cell Activation Elicited by Chimeric Exosome‐Based Nanovaccine

To assess the direct T‐cell immunostimulatory response triggered by self‐presentation of DT‐Exos, the splenocytes were prepared by grinding the spleen tissues of female C57BL/6 mice (6–8 weeks old) through 70‐µm cell strainers, before incubation with RBC lysis solution (BioLegend). Cells were then stained with the CFSE Cell Proliferation Kit (Invitrogen) according to the standard protocol. CFSE‐stained cells were seeded at a density of 1 000 000 cells well^−1^ in a 6‐well plate and exposed to the indicated exosome formulations (including T‐Exos, D‐Exos, and DT‐Exos) at equivalent dosages of 40 µg mL^−1^. After a 72 h incubation, the splenic cells were stained with fluorescently labeled antibodies against CD3 (BioLegend; 17A2) and CD8a (BioLegend; 53–6.7) for 30 min at 4 °C, and subject to flow cytometric analysis. For further evaluation of direct CD8^+^ T‐cell activation, splenocytes were plated at a density of 1 000 000 cells well^−1^ in a 6‐well plate and treated with the identical formulations as described before for 48 h. Then, cells were stained with anti‐CD3 (BioLegend; 17A2) and anti‐CD8a (BioLegend; 53–6.7) for subsequent flow cytometric assessment.

To determine the indirect DC‐to‐T immune‐activated route, well‐differentiated BMDCs were seeded at a density of 500 000 cell well^−1^ in a 6‐well plate and incubated with T‐Exos, D‐Exos, and DT‐Exos at equal dosages of 40 µg mL^−1^ for 48 h. These exosome‐treated BMDCs were subject to flow cytometric analysis of DC maturation, through staining with anti‐CD11c (BioLegend; N418), anti‐CD80 (BioLegend; 16‐10A1), and anti‐CD86 (BioLegend; GL‐1). For assessment of DC‐mediated cross‐presentation, a GL261‐OVA cell line was introduced for these OVA‐containing nanovesicles. After 48 h incubation with these aforementioned exosome formulations, BMDCs were stained with anti‐CD11c (BioLegend; N418) and anti‐SIINFEKL‐H‐2Kb (BioLegend; 25‐D1.16) for 30 min at 4 °C prior to flow cytometric detection. To evaluate the cytotoxic CD8^+^ T‐cell proliferation, BMDCs were likewise pre‐treated with T‐Exos, D‐Exos, and DT‐Exos at equal dosages of 40 µg mL^−1^ for 48 h, and underwent a 72 h co‐incubation with CFSE‐labeled splenocytes at a ratio of 1:20. After exposure to anti‐CD3 (BioLegend; 17A2) and anti‐CD8a (BioLegend; 53–6.7), cytotoxic CD8^+^ T‐cell priming capacity was identified through flow cytometric analysis. For the investigation of T‐cell tumoricidal activity, the exosome‐stimulated BMDCs were prepared as depicted above‐mentioned, and the DC‐to‐T cell co‐culture system was set up with a ratio of 1:20. 48 h after co‐incubation, the cell supernatants in the co‐culture system were collected for quantitative analysis of secreted proinflammatory cytokine IFN‐γ according to the instruction of ELISA kit (4A Biotech). In addition, activated splenocytes in this DC‐to‐T cell co‐culture system were exposed to co‐incubation with GL261 cells with a ratio of 10:1 for 24 h, and then this cytotoxicity assay was implemented using an LDH release assay kit (Beyotime).

For determination of STING activation triggered by this DT‐Exo‐STING nanovaccine, well‐differentiated BMDCs were seeded at a density of 500 000 cells well^−1^ in a 6‐well plate and exposed to the designated cGAMP‐containing formulas at dosage of cGAMP molecules (100 ng mL^−1^) for 24 h. After that, cells were stained with antibodies against p‐IRF3 (Cell Signaling Technology) according to the intranuclear staining protocol for flow cytometric evaluation. All flow cytometric data were obtained on flow cytometry (BD Accurit C6 Plus) and analyzed using FlowJo software.

### Intracranial GBM Model

For the establishment of murine orthotopic GBM models, female C57BL/6 mice (6–8 weeks old) were implanted with GL261 cells (50 000 cells per mouse) stereotactically into the left striatum of the brain using a 10‐µL Hamilton syringe and Vetbond (3M) adhesive was employed for wound closure. The coordinate of the injection site was as follows: 0.5 mm anterior, 2 mm left lateral from bregma, and 3.5 mm depth.^[^ ^51^ ^]^

### In Vivo Tissue Tropism of DT‐Exo‐STING

To assess the BBB‐crossing capacity of this chimeric nanocarrier, GL261‐bearing mice were subcutaneously administrated with cdGMP‐Dy547 alone and cdGMP‐Dy547–loaded DT‐Exos at dosage of cdGMP‐Dy547 (10 µg per mouse), and in vivo fluorescence imaging was conducted at various points of execution. After a period of 24 h, visualized analysis was further performed by leveraging surfactant assistant tissue clearing technique, namely CLARITY. The CLARITY procedure was performed as the previously reported method.^[^ ^54^ ^]^ Briefly, mice were euthanized and subject to perfusion treatment with icy PBS buffer, containing 0.5% (w/v) sodium nitrite (Sigma‐Aldrich) and 10 U mL^−1^ heparin (MedChemExpress). Then, the collected brain tissues were immersed in 4% (w/v) paraformaldehyde (BioLegend) for 2 h. For permission of adequate infiltration with monomers and initiators, brain tissues were soaked in PBS buffer comprising 4% (w/v) acrylamide (Sigma‐Aldrich) and 0.25% (w/v) 2,2′‐azobis[2‐(2‐imidazolin‐2‐yl)propane] dihydrochloride (Wako Pure Chemical Industries) for 1 day. Under the protection of nitrogen, the polymerization reaction of polyacrylamide hydrogel was triggered by maintaining this sealing system warm at 37 °C for 3 h. The obtained tissue/hydrogel hybrids were immersed in PBS solution containing 10% (w/v) sodium dodecyl sulfate (Sigma‐Aldrich) and 0.01% (w/v) sodium azide (Sigma‐Aldrich) on a water bath shaker at 37 °C for at least 3 days till these tissue/hydrogel hybrids tended toward transparency. After that, the z‐stack images were observed using the CLSM (Leica Microsystems). To investigate the influencing factors interrelated with BBB‐crossing capacity of DT‐Exos, GBM‐burdened mice underwent a subcutaneous administration with the designated cdGMP‐Dy547–containing vesicles, including nanovesicles prepared from RBCs,^[^ ^55^ ^]^ DC‐tumor chimeric micro‐sized vesicles, T‐Exos, D‐Exos and DT‐Exos, at equivalent dosages of cdGMP‐Dy547 (10 µg per mouse). 24 h period post‐administration, the brain tissues, and cervical LNs were also harvested for further immunofluorescence staining.

### DT‐Exo‐STING–Triggered Anti‐Tumor Efficacy in an Orthotopic GBM Mouse Model

After intracranial implantation with GL261‐OVA‐Luc cells for 10 days, mice were subcutaneously vaccinated with PBS solution, free cGAMP, individual DT‐Exos, T‐Exo‐STING, D‐Exo‐STING, and DT‐Exo‐STING at equivalent dosages of cGAMP molecules (10 µg per mouse) four times total, spaced 4 days apart. 24 h after the second dose, cervical LNs, and brain tumor tissues were harvested for further flow cytometry analysis. To this end, both cervical LNs and brain tumor tissues were dissociated with collagenase type IV (1 mg mL^−1^; Shanghai Yuanye Bio‐Technology), deoxyribonuclease I (0.2 mg mL^−1^; Shanghai Yuanye Bio‐Technology), and hyaluronidase (0.1 mg mL^−1^; Shanghai Yuanye Bio‐Technology) for at least 30 min. Afterward, the mixture of tissues was ground through 70‐µm cell strainers and treated with RBC lysis solution for single‐cell suspensions, before incubation with anti‐CD16/32 (BioLegend; 93) for 30 min at 4 °C. Cells were diluted to a concentration of 1 × 10^7^ cells mL^−1^ for staining with corresponding fluorescently‐labeled antibodies. For analysis of DC maturation, cell suspensions derived from cervical LNs were stained with anti‐CD11c (BioLegend; N418), anti‐CD80 (BioLegend; 16‐10A1) and anti‐CD86 (BioLegend; GL‐1). Cells within cervical LNs were subject to staining with anti‐CD11c (BioLegend; N418) and anti‐SIINFEKL‐H‐2Kb (BioLegend; 25‐D1.16) for evaluation of DC‐mediated cross‐presentation. Furthermore, tumor tissue‐derived cell samples were stained with several panels of antibodies against various T‐cell‐related markers. For identification of T‐cell differentiation, cells were stained with anti‐CD3 (BioLegend; 17A2), anti‐CD4 (BioLegend; GK1.5) and anti‐CD8a (BioLegend; 53–6.7) for 30 min at 4 °C before flow cytometric assessment. To further determine tumor antigen‐specific T‐cell response, cells stained with antibodies against CD3 (BioLegend; 17A2) and CD8a (BioLegend; 53–6.7) were subject to H‐2Kb/SIINFEKL tetramer (BioLegend) staining abiding by the manufacturer's instruction or anti‐IFN‐γ (BioLegend; XMG1.2) labeling according to the intracellular staining protocol. T‐cell proliferation was then detected through staining with anti‐CD3 (BioLegend; 17A2), anti‐CD8a (BioLegend; 53–6.7), and anti‐Ki67 (BioLegend; 16A8) following the intranuclear staining protocol. All flow cytometric data were obtained on flow cytometry (BD Accurit C6 Plus) and analyzed using FlowJo software. In addition, intracranial glioma growth was monitored by an IVIS spectrum imaging system (Perkin‐Elmer).

### Personalized Exosome‐Based Nanovaccine in Synergy with ICB Therapy for GBM Post‐Surgical Treatment

On day 10 after intracranial inoculation with GL261‐Luc cells, surgical removal of the visible tumors was conducted under a stereoscope. Bulk of tumor tissues from each GBM‐bearing mouse was resected for customized chimeric exosome‐based nanovaccine as described before, while the tiny intracranial focus was preserved to emulate an actual clinical scenario of postoperative residual microtumor sites. 4‐day period post‐operation, mice were dosed with PBS buffer, anti‐PD‐1 (BioXcell) alone, individual DT‐Exo‐STING and the combinatorial therapy (DT‐Exo‐STING + anti‐PD‐1) at equal dosages of cGAMP molecules (subcutaneous injection of 10 µg per mouse) and anti‐PD‐1 (intraperitoneal administration of 100 µg per mouse) four times total, 4 days apart. 24 h after the second vaccination, the peripheral blood of mice was collected and supplemented with RBC lysis solution for single‐cell suspensions, before incubation with anti‐CD16/32 (BioLegend; 93). After dilution to a concentration of 1 × 10^7^ cells mL^−1^, cells were subject to flow cytometric analysis of memory T‐cell response, with staining of antibodies against CD8a (BioLegend; 53–6.7), CD44 (BioLegend; IM7) and CD62L (BioLegend; MEL‐14) for 30 min at 4 °C. Flow cytometric data were acquired on flow cytometry (BD Accurit C6 Plus) and analyzed utilizing FlowJo software. In addition, recrudescent glioma burdens were monitored by an IVIS spectrum imaging system (Perkin‐Elmer).

### T‐Cell Blocking Assay

Mice bearing intracranial GL261‐Luc tumors underwent postoperative combinatorial immunotherapy (DT‐Exo‐STING + anti‐PD‐1) as described before. To validate the significance of cytotoxic CD8^+^ T cells in this antineoplastic immune response, mice were intraperitoneally injected with anti‐CD8a (BioLegend; 200 µg per mouse) for CD8^+^ T‐cell depletion or mouse IgG (Southern Biotechnology; 200 µg per mouse) as a control total 4 times, spaced 5 days apart. On day 13 after intracranial GBM inoculation, the peripheral blood of mice was collected to verify the effective blockade of CD8^+^ T cells using flow cytometry (BD Accurit C6 Plus). Intracranial tumor recrudesce was also monitored by an IVIS spectrum imaging system (Perkin‐Elmer).

### Statistical Analysis

Quantitative data were presented as means ± SD. One‐way or two‐way ANOVA with Tukey test was applied for multiple comparisons. Survivorship curves were evaluated with log‐rank Mantel–Cox test. In all cases, differences with *p <* 0.05 were deemed to be statistically significant. In specific, significance was defined as *p < 0.05, **p < 0.01, ***p < 0.001, and ****p < 0.0001. Statistical analysis was executed using GraphPad Prism7.

## Conflict of Interest

The authors declare no conflict of interest.

## Supporting information

Supporting Information

## Data Availability

The data that support the findings of this study are available from the corresponding author upon reasonable request.

## References

[1] A. Ribas , J. Wolchok , Science 2018, 359, 1350. 29567705 10.1126/science.aar4060 PMC7391259

[2] L. Chen , H. Qin , R. Zhao , X. Zhao , L. Lin , Y. Chen , Y. Lin , Y. Li , Y. Qin , Y. Li , S. Liu , K. Cheng , H. Chen , J. Shi , G. Anderson , Y. Wu , Y. Zhao , G. Nie , Sci. Transl. Med. 2021, 13, c2816. 10.1126/scitranslmed.abc2816 34233949

[3] C. Liu , X. Liu , X. Xiang , X. Pang , S. Chen , Y. Zhang , E. Ren , L. Zhang , X. Liu , P. Lv , X. Wang , W. Luo , N. Xia , X. Chen , G. Liu , Nat. Nanotechnol. 2022, 17, 531. 35410368 10.1038/s41565-022-01098-0

[4] S. Stevanovic , A. Pasetto , S. Helman , J. Gartner , T. Prickett , B. Howie , H. Robins , P. Robbins , C. Klebanoff , S. Rosenberg , C. Hinrichs , Science 2017, 356, 200. 28408606 10.1126/science.aak9510 PMC6295311

[5] P. Coulie , B. Van Den Eynde , P. Van Der Bruggen , T. Boon , Nat. Rev. Cancer 2014, 14, 135. 24457417 10.1038/nrc3670

[6] C. Melief , S. Van Der Burg , Nat. Rev. Cancer 2008, 8, 351. 18418403 10.1038/nrc2373

[7] D. Speiser , D. Liénard , N. Rufer , V. Rubio‐Godoy , D. Rimoldi , F. Lejeune , A. Krieg , J. Cerottini , P. Romero , J. Clin. Invest. 2005, 115, 739. 15696196 10.1172/JCI23373 PMC546459

[8] R. Kuai , L. Ochyl , K. Bahjat , A. Schwendeman , J. Moon , Nat. Mater. 2017, 16, 489. 28024156 10.1038/nmat4822 PMC5374005

[9] S. Liu , Q. Jiang , X. Zhao , R. Zhao , Y. Wang , Y. Wang , J. Liu , Y. Shang , S. Zhao , T. Wu , Y. Zhang , G. Nie , B. Ding , Nat. Mater. 2021, 20, 421. 32895504 10.1038/s41563-020-0793-6

[10] P. Srivastava , Curr. Opin. Immunol. 2006, 18, 201. 16464565 10.1016/j.coi.2006.01.009

[11] L. Lybaert , S. Lefever , B. Fant , E. Smits , B. De Geest , K. Breckpot , L. Dirix , S. Feldman , W. Van Criekinge , K. Thielemans , S. Van Der Burg , P. Ott , C. Bogaert , Cancer Cell 2023, 41, 15. 36368320 10.1016/j.ccell.2022.10.013

[12] S. Vardhana , M. Hwee , M. Berisa , D. Wells , K. Yost , B. King , M. Smith , P. Herrera , H. Chang , A. Satpathy , M. Van Den Brink , J. Cross , C. Thompson , Nat. Immunol. 2020, 21, 1022. 32661364 10.1038/s41590-020-0725-2 PMC7442749

[13] B. Zuo , H. Qi , Z. Lu , L.u Chen , B.o Sun , R. Yang , Y. Zhang , Z. Liu , X. Gao , A. You , L.i Wu , R. Jing , Q. Zhou , H. Yin , Nat. Commun. 2020, 11, 1790. 32286296 10.1038/s41467-020-15569-2 PMC7156382

[14] C. Gong , X. Zhang , M. Shi , F. Li , S. Wang , Y. Wang , Y. Wang , W. Wei , G. Ma , Adv. Sci. 2021, 8, 2002787. 10.1002/advs.202002787 PMC8132050 34026432

[15] J. Wolfers , A. Lozier , G. Raposo , A. Regnault , C. Théry , C. Masurier , C. Flament , S. Pouzieux , F. Faure , T. Tursz , E. Angevin , S. Amigorena , L. Zitvogel , Nat. Med. 2001, 7, 297. 11231627 10.1038/85438

[16] A. Becker , B. Thakur , J. Weiss , H. Kim , H. Peinado , D. Lyden , Cancer Cell 2016, 30, 836. 27960084 10.1016/j.ccell.2016.10.009 PMC5157696

[17] R. Kalluri , V. Lebleu , Science 2020, 367, u6977. 10.1126/science.aau6977 PMC7717626 32029601

[18] C. Théry , L. Zitvogel , S. Amigorena , Nat. Rev. Immunol. 2002, 2, 569. 12154376 10.1038/nri855

[19] S. El Andaloussi , I. Mäger , X. Breakefield , M. Wood , Nat. Rev. Drug Discovery 2013, 12, 347. 23584393 10.1038/nrd3978

[20] J. Gong , D. Chen , M. Kashiwaba , D. Kufe , Nat. Med. 1997, 3, 558. 9142127 10.1038/nm0597-558

[21] S. Koido , E. Hara , S. Homma , A. Torii , Y. Toyama , H. Kawahara , M. Watanabe , K. Yanaga , K. Fujise , H. Tajiri , J. Gong , G. Toda , Clin. Cancer Res. 2005, 11, 7891. 16278414 10.1158/1078-0432.CCR-05-1330

[22] W. Liu , M. Zou , T. Liu , J. Zeng , X. Li , W. Yu , C. Li , J. Ye , W. Song , J. Feng , X. Zhang , Nat. Commun. 2019, 10, 3199. 31324770 10.1038/s41467-019-11157-1 PMC6642123

[23] D. Wang , M. Xue , J. Chen , H. Chen , J. Liu , Q. Li , Y. Xie , Y. Hu , Y. Ni , Q. Zhou , Biomaterials 2021, 278, 121161. 34601198 10.1016/j.biomaterials.2021.121161

[24] S. Wang , F. Li , T. Ye , J. Wang , C. Lyu , S. Qing , Z. Ding , X. Gao , R. Jia , D.i Yu , J. Ren , W. Wei , G. Ma , Sci. Transl. Med. 2021, 13, b6981. 10.1126/scitranslmed.abb6981 34644149

[25] J. Zhang , B. Fan , G. Cao , W. Huang , F. Jia , G. Nie , H. Wang , Adv. Mater. 2022, 34, 2205950. 10.1002/adma.202205950 36217832

[26] C. Cui , K. Chakraborty , X. Tang , K. Schoenfelt , A. Hoffman , A. Blank , B. Mcbeth , N. Pulliam , C. Reardon , S. Kulkarni , T. Vaisar , A. Ballabio , Y. Krishnan , L. Becker , Nat. Nanotechnol. 2021, 16, 1394. 34764452 10.1038/s41565-021-00988-z

[27] L. Miao , L. Li , Y. Huang , D. Delcassian , J. Chahal , J. Han , Y. Shi , K. Sadtler , W. Gao , J. Lin , J. Doloff , R. Langer , D. G. Anderson , Nat. Biotechnol. 2019, 37, 1174. 31570898 10.1038/s41587-019-0247-3

[28] A. Ablasser , M. Goldeck , T. Cavlar , T. Deimling , G. Witte , I. Röhl , K. Hopfner , J. Ludwig , V. Hornung , Nature 2013, 498, 380. 23722158 10.1038/nature12306 PMC4143541

[29] Q. Chen , L. Sun , Z. Chen , Nat. Immunol. 2016, 17, 1142. 27648547 10.1038/ni.3558

[30] H. Ishikawa , Z. Ma , G. Barber , Nature 2009, 461, 788. 19776740 10.1038/nature08476 PMC4664154

[31] D. Leventhal , A. Sokolovska , N. Li , C. Plescia , S. Kolodziej , C. Gallant , R. Christmas , J. Gao , M. James , A. Abin‐Fuentes , M. Momin , C. Bergeron , A. Fisher , P. Miller , K. West , J. Lora , Nat. Commun. 2020, 11, 2739. 32483165 10.1038/s41467-020-16602-0 PMC7264239

[32] J. Liang , X. Jin , S. Zhang , Q. Huang , P. Ji , X. Deng , S. Cheng , W. Chen , X. Zhang , Sci. Bull. 2023, 68, 622. 10.1016/j.scib.2023.02.027 36914548

[33] X. Li , S. Khorsandi , Y. Wang , J. Santelli , K. Huntoon , N. Nguyen , M. Yang , D. Lee , Y. Lu , R. Gao , B. Kim , C. De Gracia Lux , R. Mattrey , W. Jiang , J. Lux , Nat. Nanotechnol. 2022, 17, 891. 35637356 10.1038/s41565-022-01134-z PMC9378430

[34] P. Bao , Z. Zheng , J. Ye , X. Zhang , Nano Lett. 2022, 22, 2217. 35254071 10.1021/acs.nanolett.1c03996

[35] D. Shae , K. Becker , P. Christov , D. Yun , A. Lytton‐Jean , S. Sevimli , M. Ascano , M. Kelley , D. Johnson , J. Balko , J. Wilson , Nat. Nanotechnol. 2019, 14, 269. 30664751 10.1038/s41565-018-0342-5 PMC6402974

[36] Y. Liu , W. Crowe , L. Wang , Y. Lu , W. Petty , A. Habib , D. Zhao , Nat. Commun. 2019, 10, 5108. 31704921 10.1038/s41467-019-13094-5 PMC6841721

[37] I. Herrmann , M. Wood , G. Fuhrmann , Nat. Nanotechnol. 2021, 16, 748. 34211166 10.1038/s41565-021-00931-2

[38] W. Liu , M. Zou , T. Liu , J. Zeng , X. Li , W. Yu , C. Li , J. Ye , W. Song , J. Feng , X. Zhang , Adv. Mater. 2019, 31, 1900499. 10.1002/adma.201900499 30907473

[39] D. You , G. Lim , S. Kwon , W. Um , B. Oh , S. Song , J. Lee , D. Jo , Y. Cho , J. Park , Sci. Adv. 2021, 7, e83. 10.1126/sciadv.abe0083 PMC8172131 34078596

[40] K. Popowski , A. Moatti , G. Scull , D. Silkstone , H. Lutz , B. Abad , A. George , E. Belcher , D. Zhu , X. Mei , X. Cheng , M. Cislo , A. Ghodsi , Y. Cai , K. Huang , J. Li , A. Brown , A. Greenbaum , P. Dinh , K. Cheng , Matter 2022, 5, 2960. 35847197 10.1016/j.matt.2022.06.012 PMC9272513

[41] J. Van Den Boorn , M. Schlee , C. Coch , G. Hartmann , Nat. Biotechnol. 2011, 29, 325. 21478846 10.1038/nbt.1830

[42] M. Kovar , O. Boyman , X. Shen , I. Hwang , R. Kohler , J. Sprent , Proc. Natl. Acad. Sci. USA 2006, 103, 11671. 16855047 10.1073/pnas.0603466103 PMC1544228

[43] J. Ridge , F. Di Rosa , P. Matzinger , Nature 1998, 393, 474. 9624003 10.1038/30989

[44] X. Yang , Y. Yang , J. Bian , J. Wei , Z. Wang , Z. Zhou , Z. Li , M. Sun , Nano Today 2021, 38, 101109.

[45] D. Torralba , F. Baixauli , C. Villarroya‐Beltri , I. Fernández‐Delgado , A. Latorre‐Pellicer , R. Acín‐Pérez , N. Martín‐Cófreces , Á. Jaso‐Tamame , S. Iborra , I. Jorge , G. González‐Aseguinolaza , J. Garaude , M. Vicente‐Manzanares , J. Enríquez , M. Mittelbrunn , F. Sánchez‐Madrid , Nat. Commun. 2018, 9, 2658. 29985392 10.1038/s41467-018-05077-9 PMC6037695

[46] J. Treweek , K. Chan , N. Flytzanis , B. Yang , B. Deverman , A. Greenbaum , A. Lignell , C. Xiao , L. Cai , M. Ladinsky , P. Bjorkman , C. Fowlkes , V. Gradinaru , Nat. Protoc. 2015, 10, 1860. 26492141 10.1038/nprot.2015.122 PMC4917295

[47] W. Tang , W. Fan , J. Lau , L. Deng , Z. Shen , X. Chen , Chem. Soc. Rev. 2019, 48, 2967. 31089607 10.1039/c8cs00805a

[48] B. Li , H. Xiao , M. Cai , X. Li , X. Xu , S. Wang , S. Huang , Y. Wang , D. Cheng , P. Pang , H. Shan , X. Shuai , Adv. Funct. Mater. 2020, 30, 1909117.

[49] G. Cheng , W. Li , L. Ha , X. Han , S. Hao , Y. Wan , Z. Wang , F. Dong , X. Zou , Y. Mao , S. Zheng , J. Am. Chem. Soc. 2018, 140, 7282. 29809001 10.1021/jacs.8b03584

[50] J. Li , J. Li , Y. Peng , Y. Du , Z. Yang , X. Qi , J. Controlled Release 2023, 353, 423. 10.1016/j.jconrel.2022.11.053 36470333

[51] J. Kuang , W. Song , J. Yin , X. Zeng , S. Han , Y. Zhao , J. Tao , C. Liu , X. He , X. Zhang , Adv. Funct. Mater. 2018, 28, 1800025.

[52] G. Chen , A. Huang , W. Zhang , G. Zhang , M. Wu , W. Xu , Z. Yu , J. Yang , B. Wang , H. Sun , H. Xia , Q. Man , W. Zhong , L. Antelo , B. Wu , X. Xiong , X. Liu , L. Guan , T. Li , S. Liu , R. Yang , Y. Lu , L. Dong , S. Mcgettigan , R. Somasundaram , R. Radhakrishnan , G. Mills , Y. Lu , J. Kim , Y. H. Chen , et al., Nature 2018, 560, 382. 30089911 10.1038/s41586-018-0392-8 PMC6095740

[53] M. Lutz , N. Kukutsch , A. Ogilvie , S. RößNer , F. Koch , N. Romani , G. Schuler , J. Immunol. Methods 1999, 223, 77. 10037236 10.1016/s0022-1759(98)00204-x

[54] D. Zheng , Y. Chen , Z. Li , L. Xu , C. Li , B. Li , J. Fan , S. Cheng , X. Zhang , Nat. Commun. 2018, 9, 1680. 29700283 10.1038/s41467-018-03233-9 PMC5920064

[55] C. Hu , L. Zhang , S. Aryal , C. Cheung , R. Fang , L. Zhang , Proc. Natl. Acad. Sci. USA 2011, 108, 10980. 21690347 10.1073/pnas.1106634108 PMC3131364

