# Supplementary material for: Chimeric Exosomes Functionalized with STING Activation for Personalized Glioblastoma Immunotherapy
Source: Adv Sci (Weinh). 2023 Dec 10;11(6):2306336. doi: 10.1002/advs.202306336 (PMC10853748; doi:10.1002/advs.202306336)
Supplement: Supplementary file 1 — Supporting Information [file ADVS-11-2306336-s001.pdf]

ADVANCED SCIENCE
Open Access

Supporting Information

for *Adv. Sci.*, DOI 10.1002/advs.202306336

Chimeric Exosomes Functionalized with STING Activation for Personalized Glioblastoma Immunotherapy

*Peng Bao, Hui-Yun Gu, Jing-Jie Ye, Jin-Lian He, Zhenlin Zhong, Ai-Xi Yu and Xian-Zheng Zhang**

Supporting Information of

Chimeric Exosomes Functionalized with STING Activation for Personalized Glioblastoma Immunotherapy

*Peng Bao, Hui-Yun Gu, Jing-Jie Ye, Jin-Lian He, Zhenlin Zhong, Ai-Xi Yu, and Xian-Zheng Zhang**

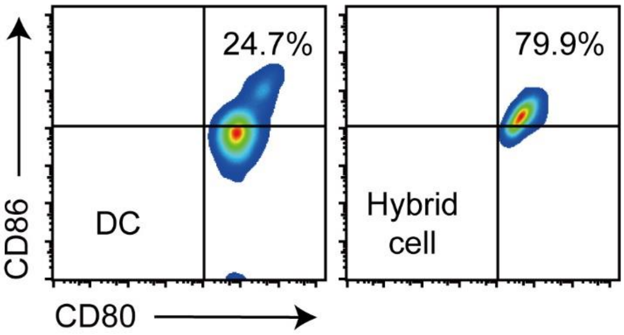

Figure S1. Flow cytometry plots to to exhibit the immunostimulatory proportions of DC maturation ($CD11c^{+}CD80^{+}CD86^{+}$).

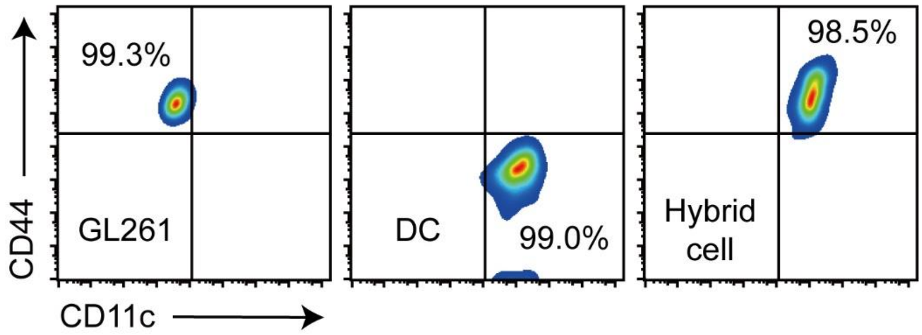

Figure S2. Flow cytometry analysis of anti-CD44–labeled GL261 cells, anti-CD11c–marked DCs, and the double-antibody–labeled DC-tumor hybrid cells.

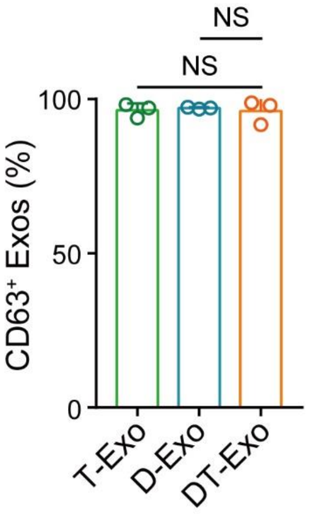

Figure S3. Flow cytometric quantitative analysis of CD63 on T-Exos, D-Exos and DT-Exos (*n* = 3; one-way ANOVA with Tukey's multiple comparisons test). Data are represented as means ± SD. NS: not significant.

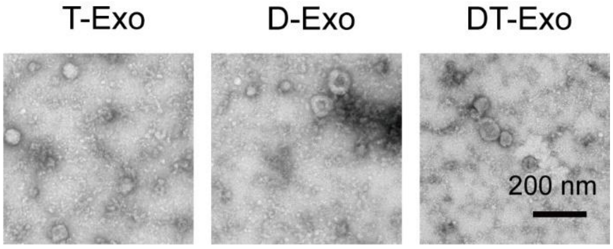

Figure S4. TEM images of T-Exos, D-Exos and DT-Exos.

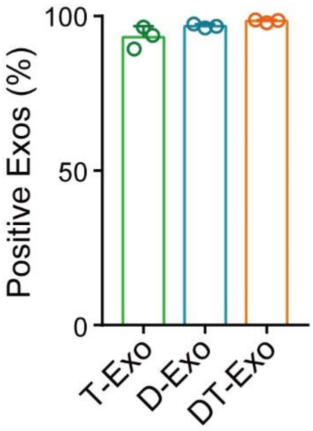

Figure S5. Flow cytometric quantitation of anti-CD44–labeled T-Exos, anti-CD11c–marked D-Exos, and the double-antibody–labeled DT-Exos (*n* = 3). Data are represented as means ± SD.

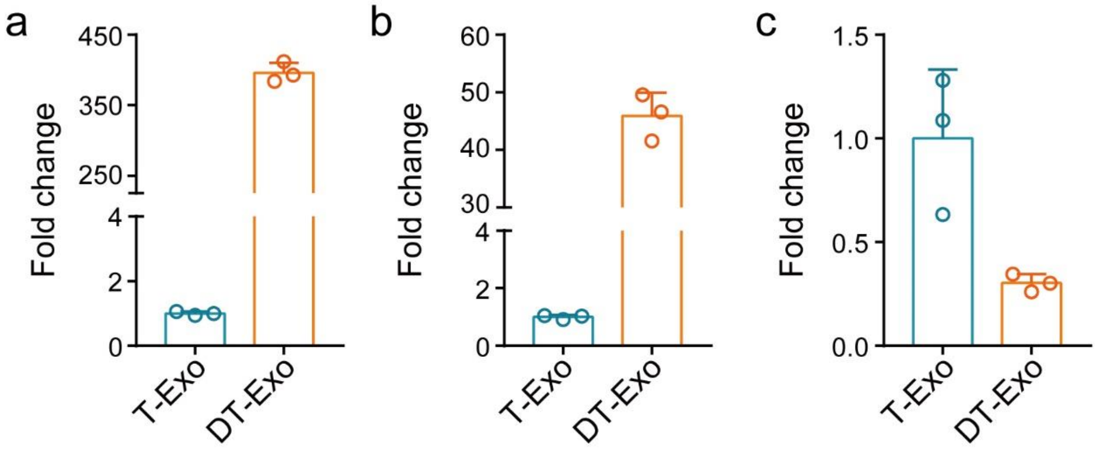

Figure S6. Quantitative PCR with reverse transcription (RT–qPCR) of exosome miRNA including *miR-211-3p* (a), *miR-155-5p* (b) and *miR-187-5p* (c) in DT-Exos versus T-Exos (*n* = 3). Data are represented as means ± SD.

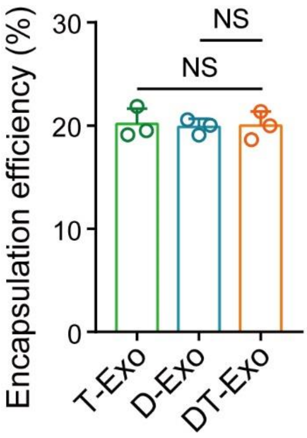

Figure S7. Encapsulation efficiencies of fluorescently-labeled CDN (cdGMP-Dy547) within T-Exos, D-Exos, and DT-Exos (n = 3; one-way ANOVA with Tukey's multiple comparisons test). Data are represented as means ± SD. NS: not significant.

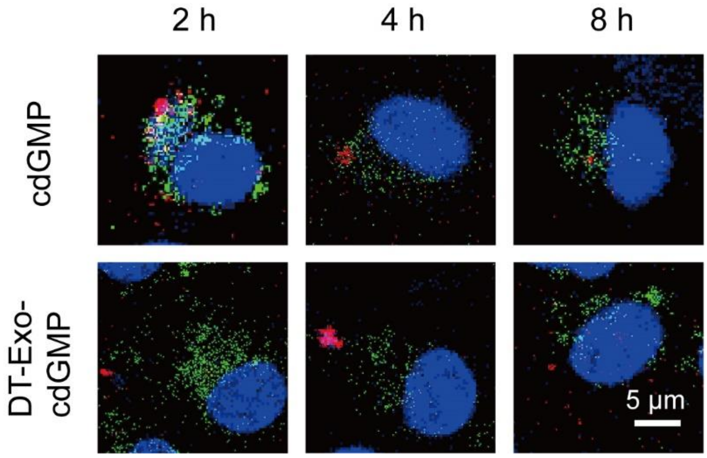

Figure S8. CLSM images of cdGMP-Dy547 distribution in BMDCs post-incubation with individual cdGMP-Dy547 and DT-Exo-cdGMP at the indicated time points. Nucleus (blue), lysosome (green) and cdGMP-Dy547 (red) in confocal images.

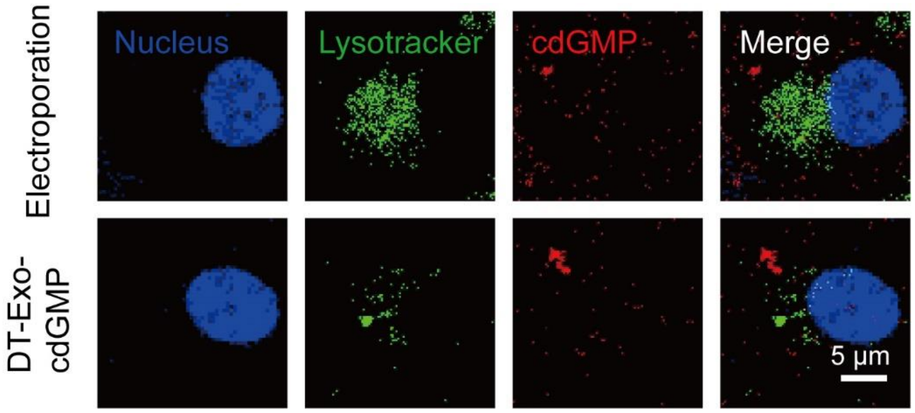

Figure S9. CLSM images of fluorescence distribution in direct electroporation-treated BMDCs with cdGMP-Dy547 and BMDCs after 4-hour incubation with cdGMP-Dy547–loaded DT-Exos. Nucleus (blue), lysosome (green) and cdGMP-Dy547 (red) in confocal images.

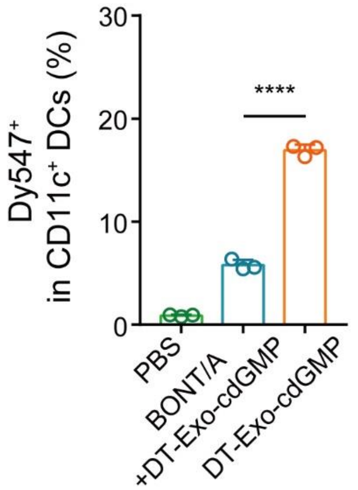

Figure S10. Flow cytometric quantitative analysis of CD11c$^+$Dy547$^+$ cells after 4-hour incubation with DT-Exo-cdGMP pretreated with Botulinum toxin serotype A (BONT/A) overnight (n = 3; one-way ANOVA with Tukey’s multiple comparisons test). Data are represented as means ± SD. ****P < 0.0001.

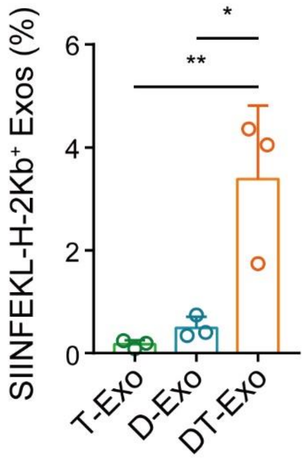

Figure S11. The percentages of SIINFEKL-H-2Kb$^+$ exosomes detected by means of flow cytometry (n = 3; one-way ANOVA with Tukey's multiple comparisons test). Data are represented as means ± SD. *P < 0.05, **P < 0.01.

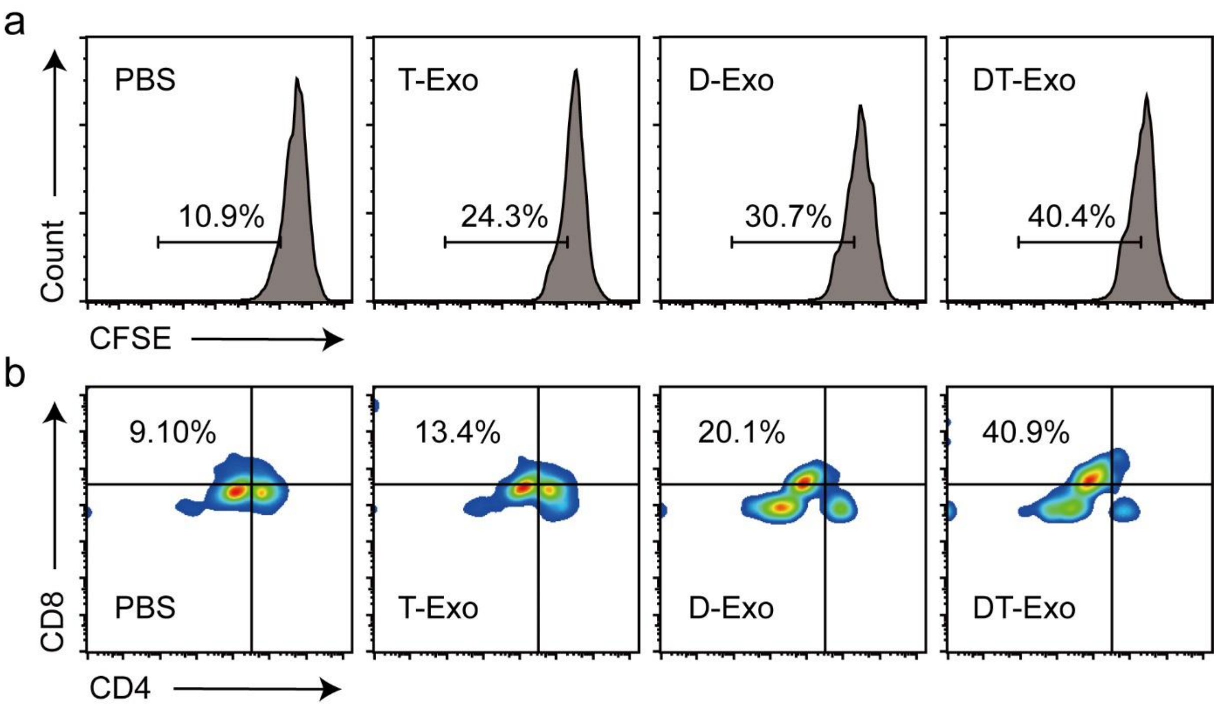

Figure S12. Representative flow cytometry plots presenting (a) splenic $CD3^+CD8^+$ T-cell proliferation with the staining of CFSE and (b) the differentiation percentages of $CD3^+CD8^+$ splenocytes after direct incubation with T-Exos, D-Exos, and DT-Exos.

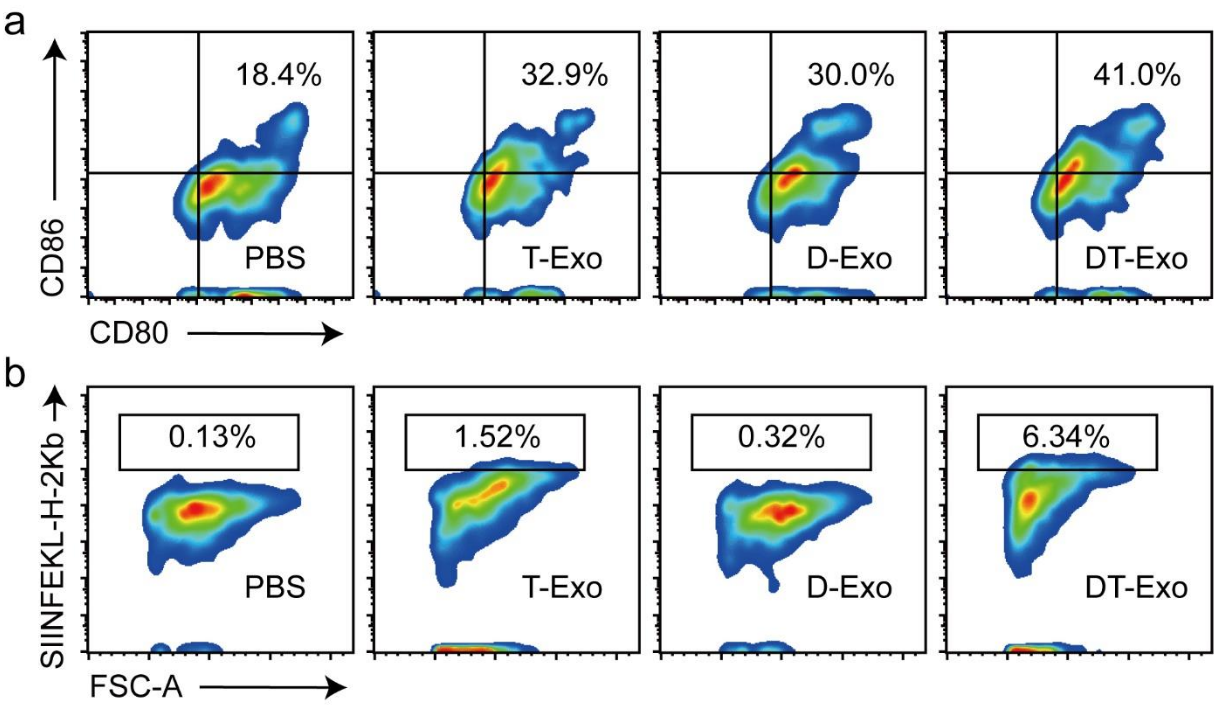

Figure S13. Representative flow cytometry data to exhibit (a) the immunostimulatory proportions of matured BMDCs ($CD11c^{+}CD80^{+}CD86^{+}$) and (b) DC-mediated cross-presentaion ($CD11c^{+}$SIINFEKL-H-$2Kb^{+}$) after treatment with the designated exosome formulations.

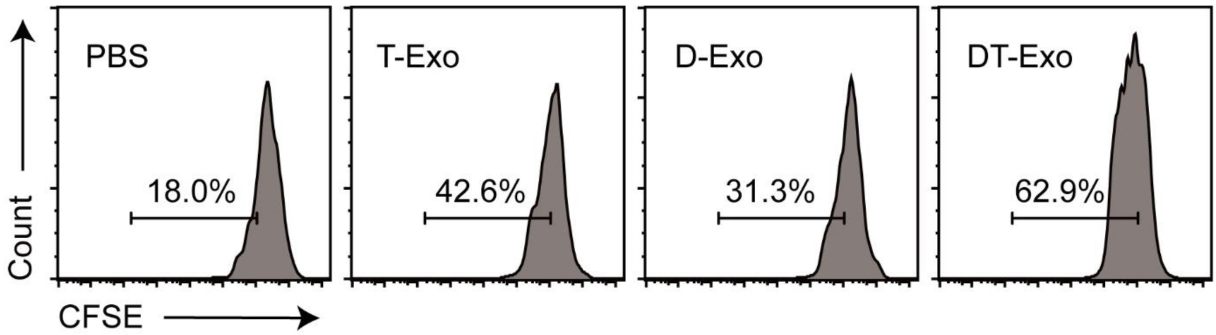

Figure S14. Flow cytometry histograms of CFSE staining in $CD3^{+}CD8^{+}$ splenocytes after incubation with exosome-stimulated BMDCs.

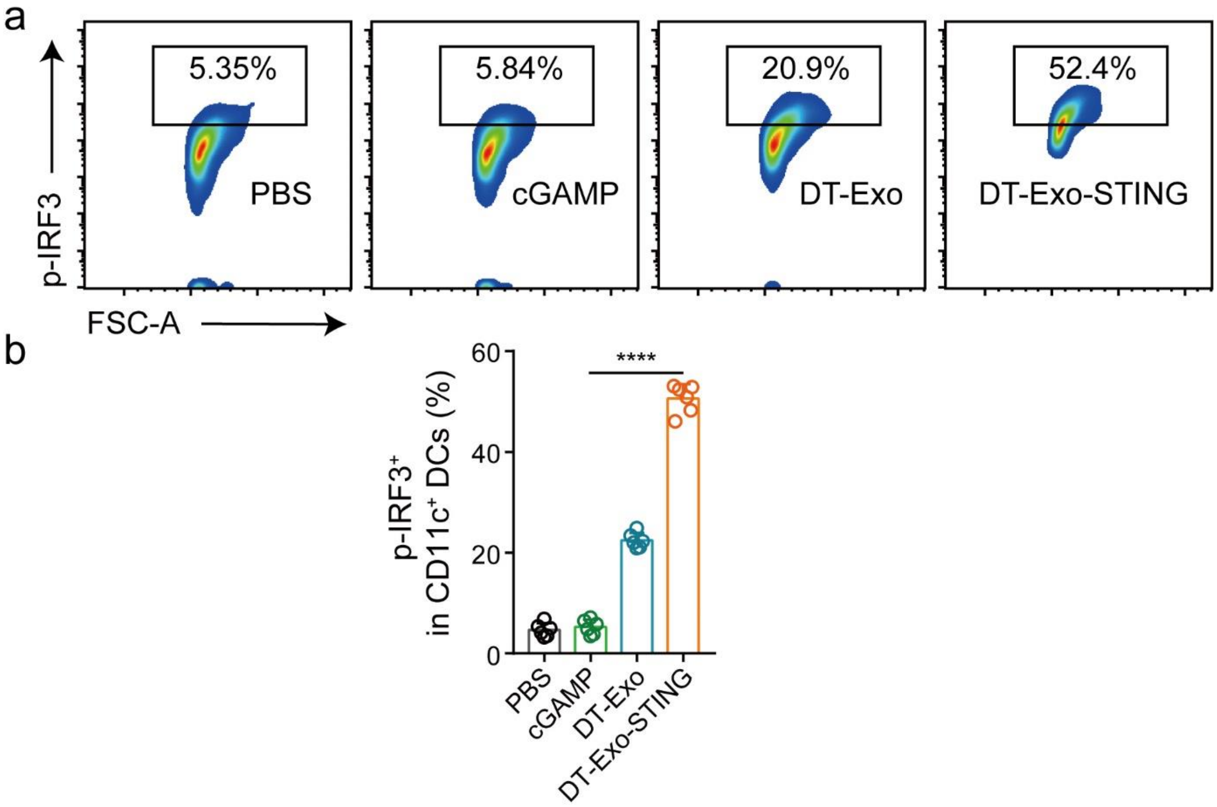

Figure S15. Representative flow cytometry data (a) and quantification (b) of p-IRF3 response elicited by 24-hour incubation with PBS, free cGAMP, DT-Exos and DT-Exo-STING in CD11c$^+$ BMDCs (n = 6; one-way ANOVA with Tukey's multiple comparisons test). Data in (b) are represented as means ± SD. ****P < 0.0001.

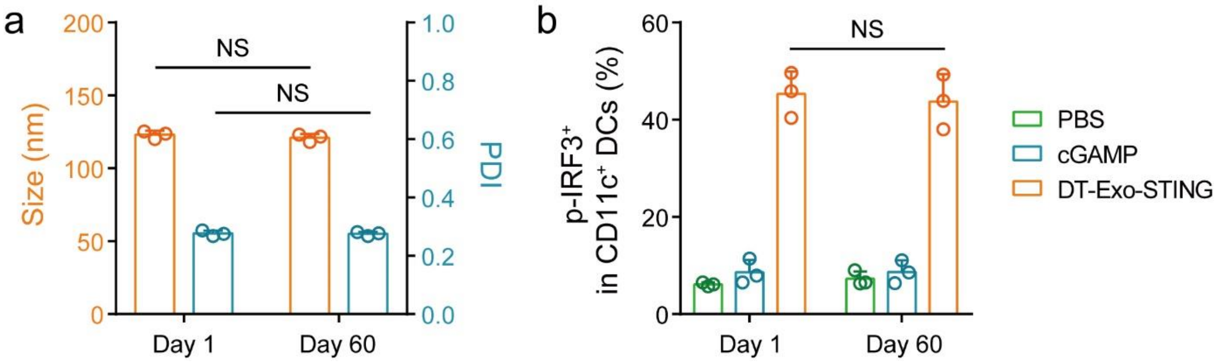

Figure S16. a) Size distributions and polydispersity index (PDI) of T-Exos, D-Exos and DT-Exos stored at the different time points (n = 3; two-way ANOVA with Tukey's multiple comparisons test). b) p-IRF3 response to the indicated cGAMP-containing formulations in CD11c$^+$ BMDCs at the different time points (n = 3; two-way ANOVA with Tukey's multiple comparisons test). Data in (a and b) are represented as means ± SD. NS: not significant.

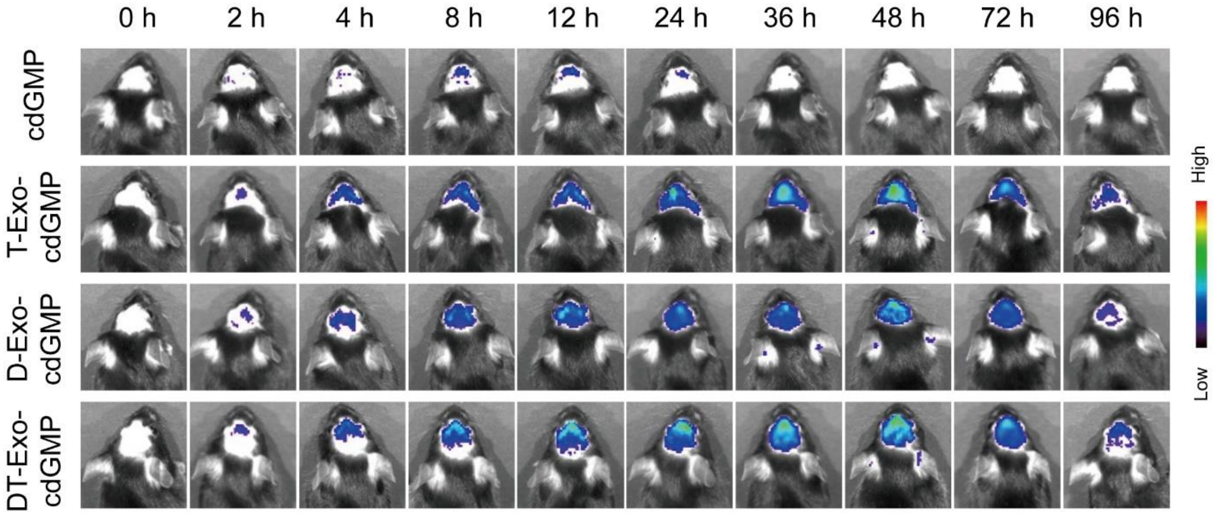

Figure S17. Representative in vivo fluorescence imaging in brain regions of healthy mice at the assigned time points post-administration of cdGMP-Dy547–containing formulations.

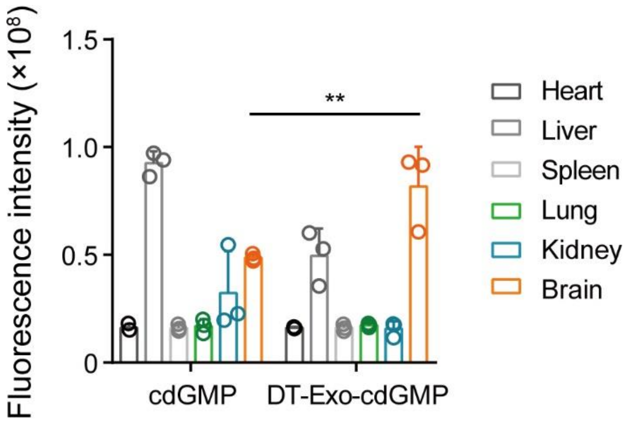

Figure S18. Quantitative analysis of ex vivo fluorescence distribution within the major organs collected from GL261 tumor-bearing mice post-administration of cdGMP-Dy547–containing formulations (n = 3; two-way ANOVA with Tukey's multiple comparisons test). Data are represented as means ± SD. **P < 0.01.

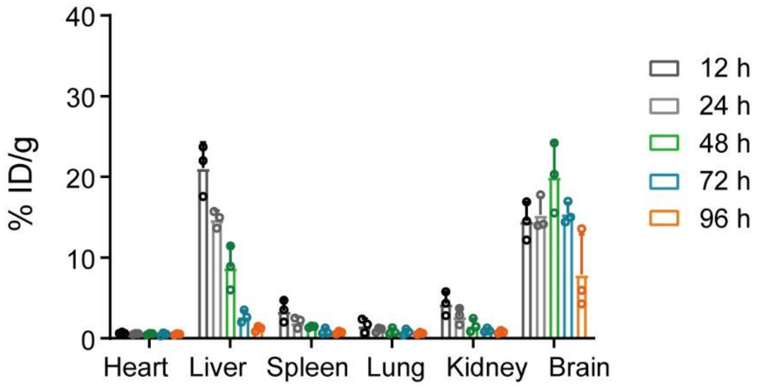

Figure S19. The percentages of injected dose per gram of tissue (% ID/g) within the major organs collected from GL261 tumor-bearing mice post-administration of DT-Exo-cdGMP (n = 3). Data are represented as means ± SD.

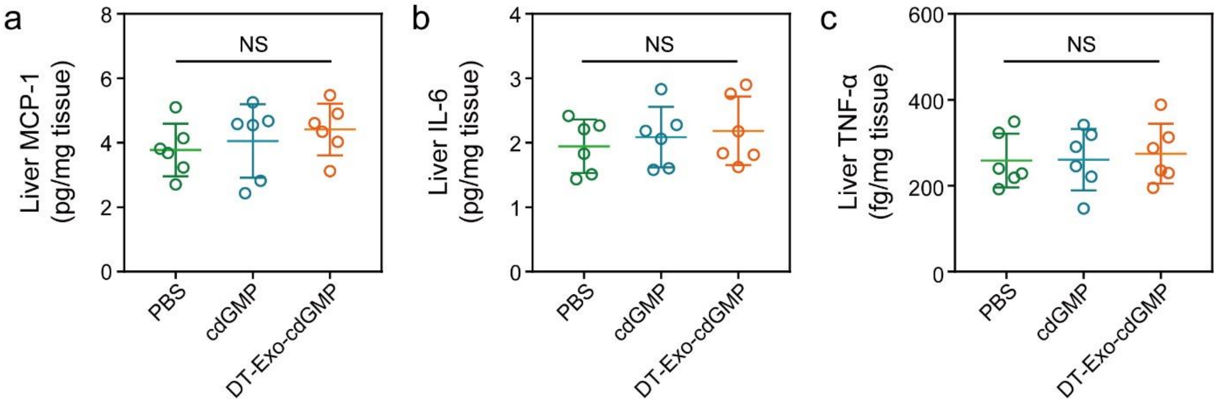

Figure S20. Pro-inflammatory cytokines including MCP-1 (a), IL-6 (b) and TNF-α (c) from the liver tissues 4 days after treatment (*n* = 6; one-way ANOVA with Tukey's multiple comparisons test). Data in (a to c) are represented as means ± SD. NS: not significant.

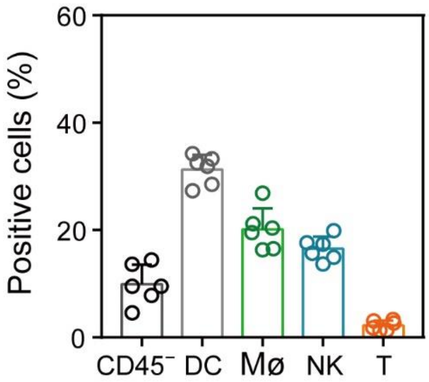

Figure S21. Proportion of cdGMP-Dy547$^+$ cells among cell populations within the tumor tissues post-administration of cdGMP-Dy547–containing formulations (*n* = 6). Data are represented as means ± SD.

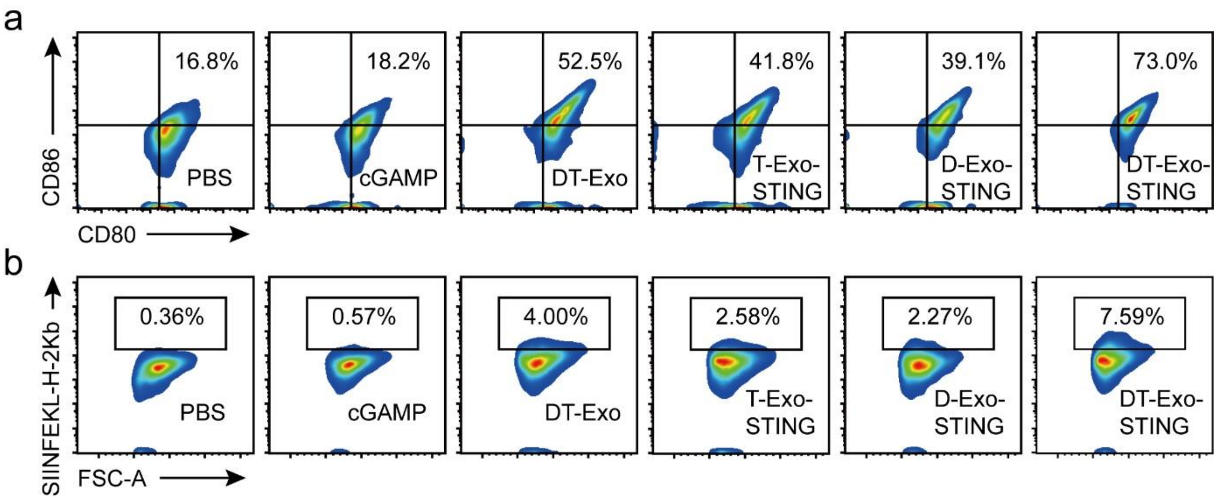

Figure S22. Representative flow cytometry plots presenting (a) the percentages of DC maturation ($CD11c^{+}CD80^{+}CD86^{+}$) and (b) the ability of DC-mediated antigen cross-presentaion ($CD11c^{+}$SIINFEKL-H-2Kb^{+}) in cervical lymph nodes of mice after subcutaneous administration with the designated cancer vaccines.

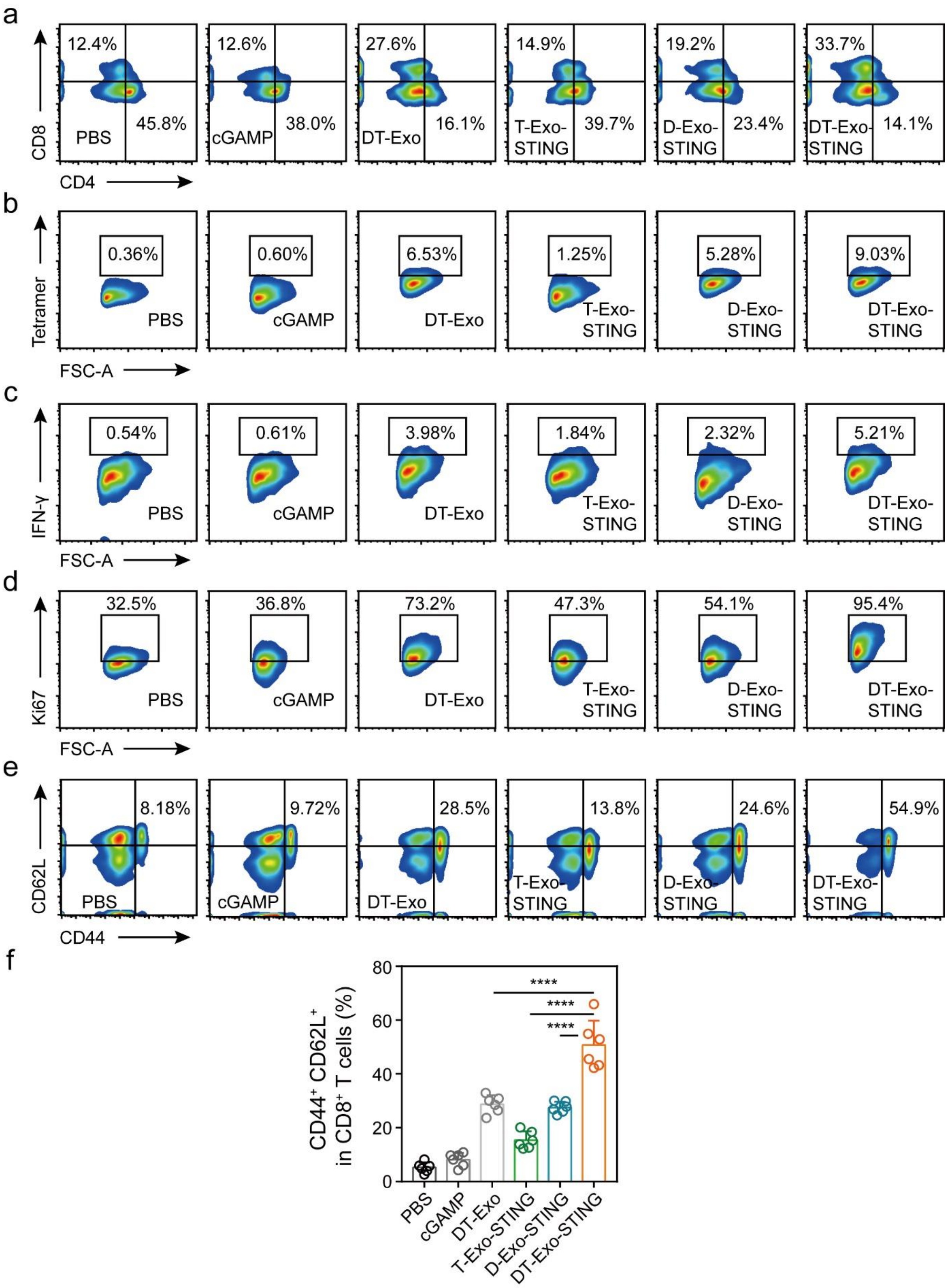

Figure S23. Representative flow cytometry data to exhibit (a) the differentiation of tumor-infiltrating CD3$^+$CD8$^+$ and CD3$^+$CD4$^+$ T lymphocytes, (b) the percentages of H-2Kb/SIINFEKL tetramer staining of CD3$^+$CD8$^+$ T cells within tumours, (c) the number of IFN-γ–positive cells in intratumoural CD3$^+$CD8$^+$ T cells, (d) the proportions of CD3$^+$CD8$^+$Ki67$^+$ T

lymphocytes infiltrated into intracranial tumour tissues and (e) the percentages of T_{CM} ($CD8^+CD44^+CD62L^+$) after various treatments. f) Flow cytometric quantitation of T_{CM} ($CD8^+CD44^+CD62L^+$) in the peripheral blood of mice administrated with the designated formulations (*n* = 6; one-way ANOVA with Tukey's multiple comparisons test). Data in (f) are represented as means ± SD. ****$P < 0.0001$.

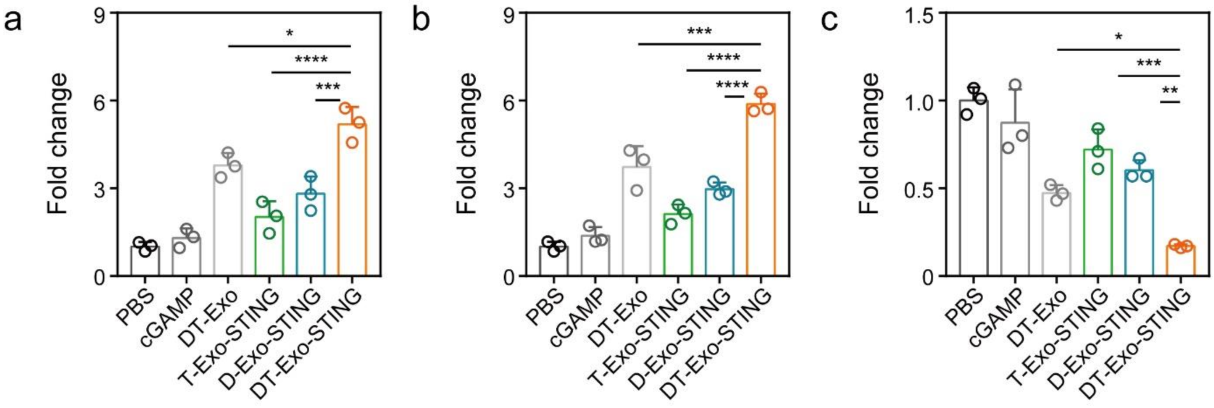

Figure S24. RT–qPCR of tumor miRNA within brain tissues including *miR-211-3p* (a), *miR-155-5p* (b) and *miR-187-5p* (c) after various treatments versus PBS group (n = 3; one-way ANOVA with Tukey's multiple comparisons test). Data in (a to c) are represented as means ± SD. *P < 0.05, **P < 0.01, ***P < 0.001, ****P < 0.0001.

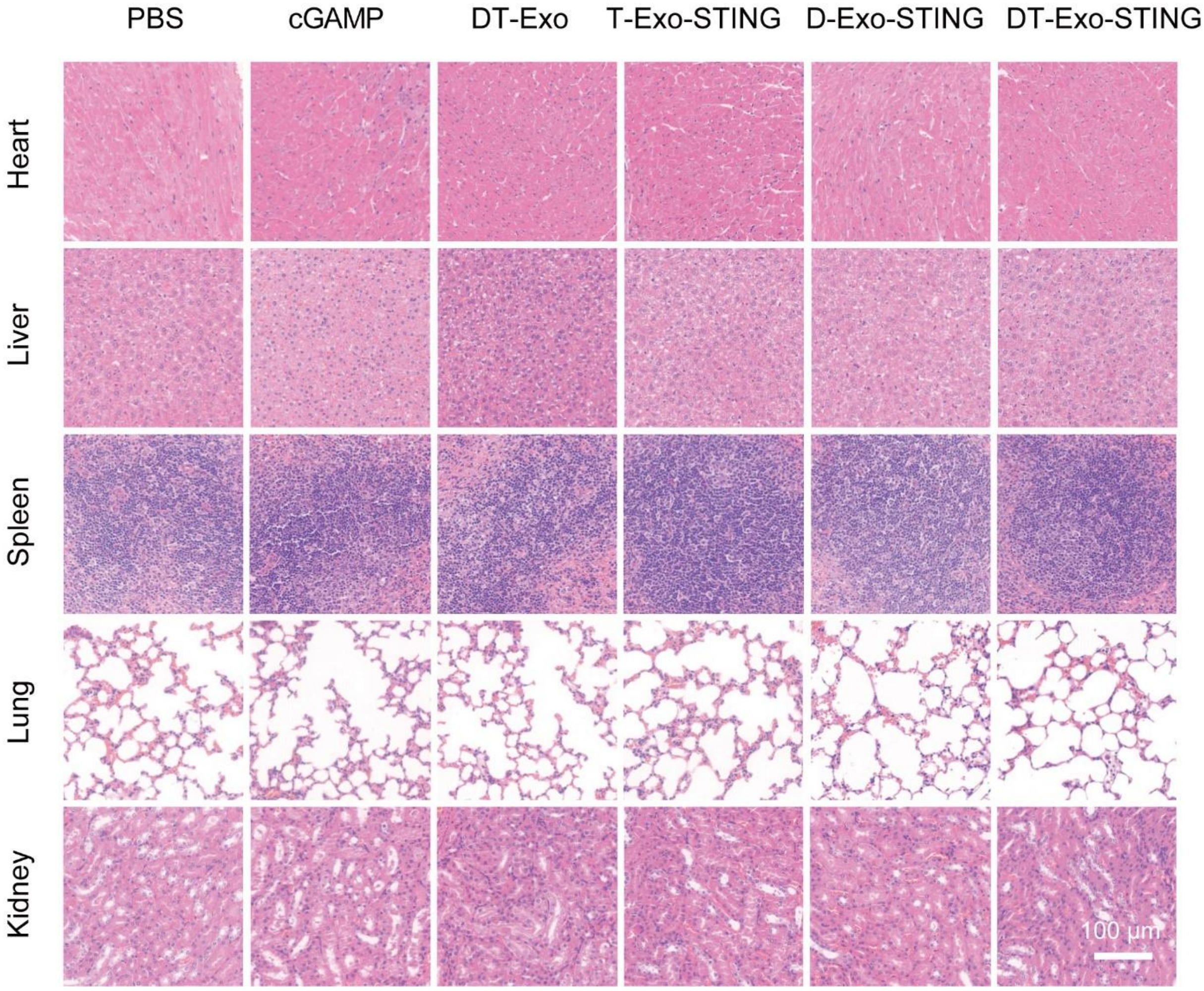

Figure S25. H&E section analysis of principal organs (heart, liver, spleen, lung, and kidney) after subcutaneous administration with the indicated vaccine formulations.

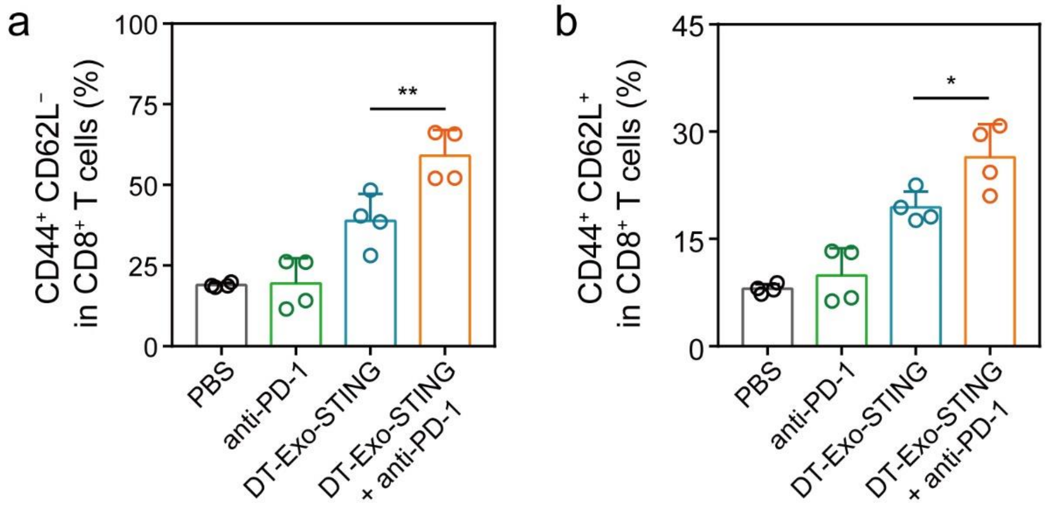

Figure S26. Flow cytometric quantitative analysis of (a) T_{EM} ($CD8^+CD44^+CD62L^−$) and (b) T_{CM} ($CD8^+CD44^+CD62L^+$) in the peripheral blood of mice administrated with the assigned formulations (n = 4; one-way ANOVA with Tukey's multiple comparisons test). Data in (a) and (b) are represented as means ± SD. *P < 0.05, **P < 0.01.

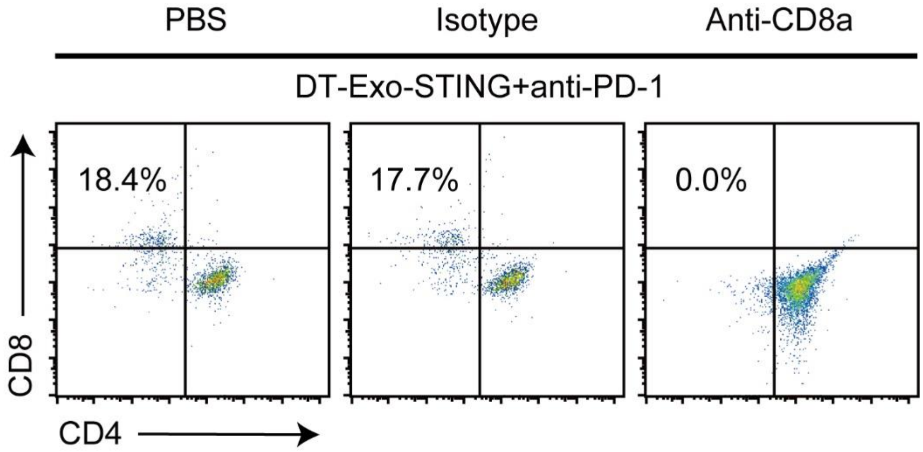

Figure S27. Flow cytometric analysis of $CD3^{+}CD8^{+}$ T cells in the peripheral blood after treatment with anti-CD8a or an isotype monoclonal antibody as control.

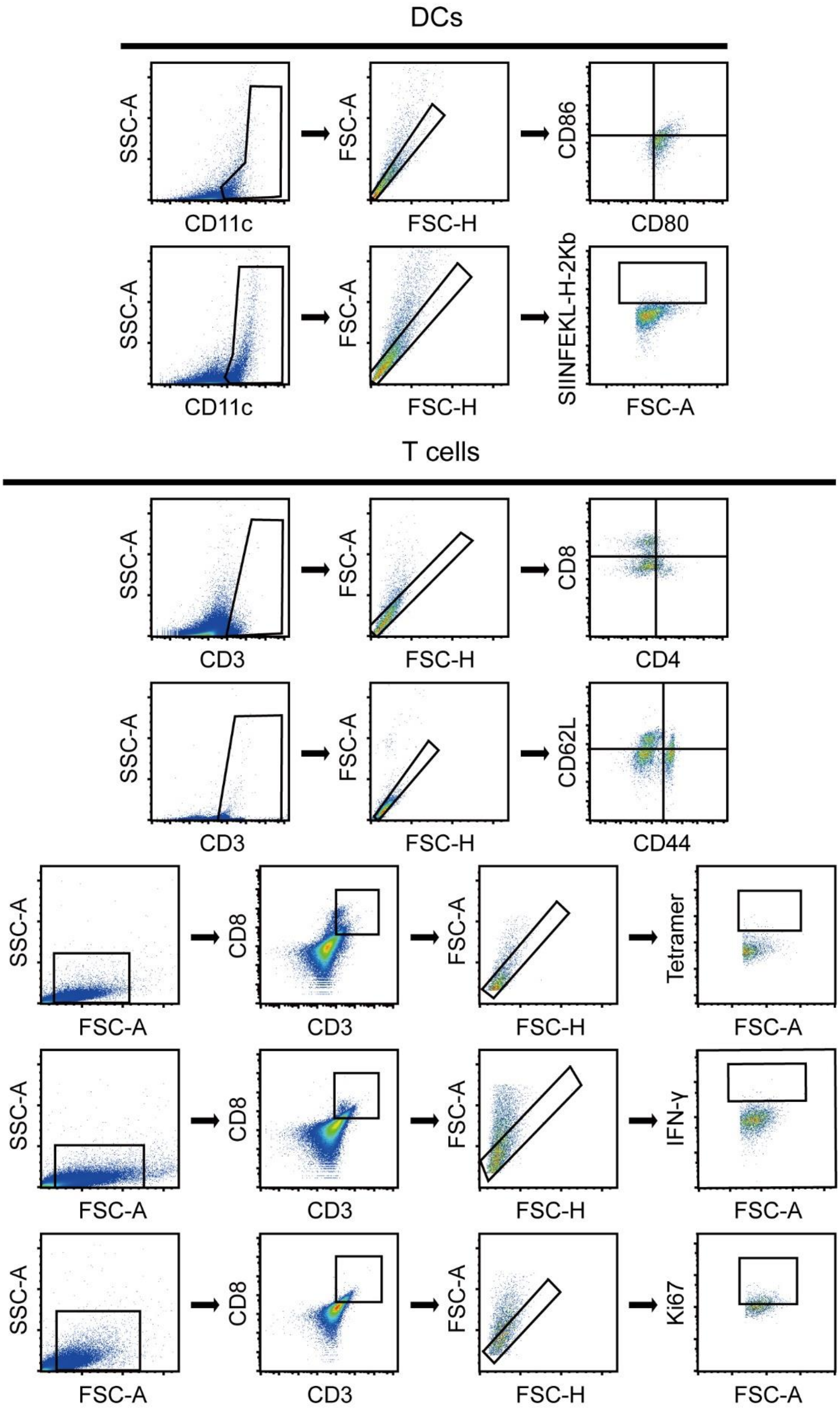

Figure S28. Flow cytometry gating strategies of immune cells. All cell statistics were conducted with the single cell format. $CD11c^{+}CD80^{+}CD86^{+}$ cells are defined as mature DCs, and $CD11c^{+}$SIINFEKL-H-2Kb^{+} cells are defined as DCs with antigen cross-presentation performance. $CD3^{+}CD8^{+}$ cells and $CD3^{+}CD4^{+}$ cells are respectively defined as cytotoxic T cells

and T helper cells. $CD8^+CD44^+CD62L^-$ cells and $CD8^+CD44^+CD62L^+$ cells are respectively defined as T_{EM} cells and T_{CM} cells. $CD3^+CD8^+$SIINFEKL-MHC-I tetramer$^+$ and $CD3^+CD8^+$IFN-γ^+ cells are defined as antigen-specific T cells. $CD3^+CD8^+Ki67^+$ cells are defined as proliferative T cells.
